# Phylogenomics provides a robust topology of the major cnidarian lineages and insights on the origins of key organismal traits

**DOI:** 10.1186/s12862-018-1142-0

**Published:** 2018-04-13

**Authors:** Ehsan Kayal, Bastian Bentlage, M. Sabrina Pankey, Aki H. Ohdera, Monica Medina, David C. Plachetzki, Allen G. Collins, Joseph F. Ryan

**Affiliations:** 1 Department of Invertebrate Zoology, National Museum of Natural History, Smithsonian Institution, Washington, DC, USA; 2 UPMC, CNRS, FR2424, ABiMS, Station Biologique, 29680 Roscoff, France; 3 Marine Laboratory, University of Guam, UOG Station, Mangilao, GU 96923 USA; 4 Department of Biology, Pennsylvania State University, University Park, PA USA; 5 Department of Molecular, Cellular and Biomedical Sciences, University of New Hampshire, Durham, NH USA; 6 National Systematics Laboratory, NOAA Fisheries, National Museum of Natural History, Smithsonian Institution, Washington, DC, USA; 7 Whitney Laboratory for Marine Bioscience, University of Florida, St Augustine, FL USA; 8 Department of Biology, University of Florida, Gainesville, FL USA

**Keywords:** Cnidaria, Genome-scale dataset, Phylogenomic analysis, Acraspeda, Staurozoa, Life history evolution

## Abstract

**Background:**

The phylogeny of Cnidaria has been a source of debate for decades, during which nearly all-possible relationships among the major lineages have been proposed. The ecological success of Cnidaria is predicated on several fascinating organismal innovations including stinging cells, symbiosis, colonial body plans and elaborate life histories. However, understanding the origins and subsequent diversification of these traits remains difficult due to persistent uncertainty surrounding the evolutionary relationships within Cnidaria. While recent phylogenomic studies have advanced our knowledge of the cnidarian tree of life, no analysis to date has included genome-scale data for each major cnidarian lineage.

**Results:**

Here we describe a well-supported hypothesis for cnidarian phylogeny based on phylogenomic analyses of new and existing genome-scale data that includes representatives of all cnidarian classes. Our results are robust to alternative modes of phylogenetic estimation and phylogenomic dataset construction. We show that two popular phylogenomic matrix construction pipelines yield profoundly different datasets, both in the identities and in the functional classes of the loci they include, but resolve the same topology. We then leverage our phylogenetic resolution of Cnidaria to understand the character histories of several critical organismal traits. Ancestral state reconstruction analyses based on our phylogeny establish several notable organismal transitions in the evolutionary history of Cnidaria and depict the ancestral cnidarian as a solitary, non-symbiotic polyp that lacked a medusa stage. In addition, Bayes factor tests strongly suggest that symbiosis has evolved multiple times independently across the cnidarian radiation.

**Conclusions:**

Cnidaria have experienced more than 600 million years of independent evolution and in the process generated an array of organismal innovations. Our results add significant clarification on the cnidarian tree of life and the histories of some of these innovations. Further, we confirm the existence of Acraspeda (staurozoans plus scyphozoans and cubozoans), thus reviving an evolutionary hypothesis put forward more than a century ago.

**Electronic supplementary material:**

The online version of this article (10.1186/s12862-018-1142-0) contains supplementary material, which is available to authorized users.

## Background

Cnidaria is a diverse phylum of mostly marine species comprised of three major clades: Anthozoa, Endocnidozoa and Medusozoa [1]. Anthozoa encompasses more than half (7200 of 13,300) of the known cnidarian species and consists of Octocorallia (sea pens, sea fans and soft corals), Hexacorallia (stony corals, black corals, sea anemones, zoantharians and corallimorpharians) and Ceriantharia (tube anemones). Endocnidozoa is an entirely parasitic clade that includes about 2200 species of Myxozoa (minute endoparasites of invertebrates and vertebrates with complex life cycles) and the monotypic Polypodiozoa (a parasite that infects the eggs of sturgeon and paddlefish). It was not until after a long line of evidence that it became clear that Myxozoa was a clade within Cnidaria (reviewed in [2, 3]). Finally, Medusozoa consists of Cubozoa (45 species of box jellyfish), Hydrozoa (3600 species of hydroids, siphonophores and hydromedusae), Scyphozoa (200 species of true jellyfish) and Staurozoa (50 species of benthic stalked jellyfish). The ecological success of Cnidaria is predicated on several fascinating organismal innovations including stinging cells called cnidocytes, relationships with phototrophic endosymbiotic eukaryotes, colonial body plans and the metagenetic life cycle that includes medusa (jellyfish) and polyp stages. However, understanding the origins and subsequent diversification of these critical innovations remains difficult due to persistent uncertainty surrounding the evolutionary relationships within Cnidaria.

**Fig. 1:**
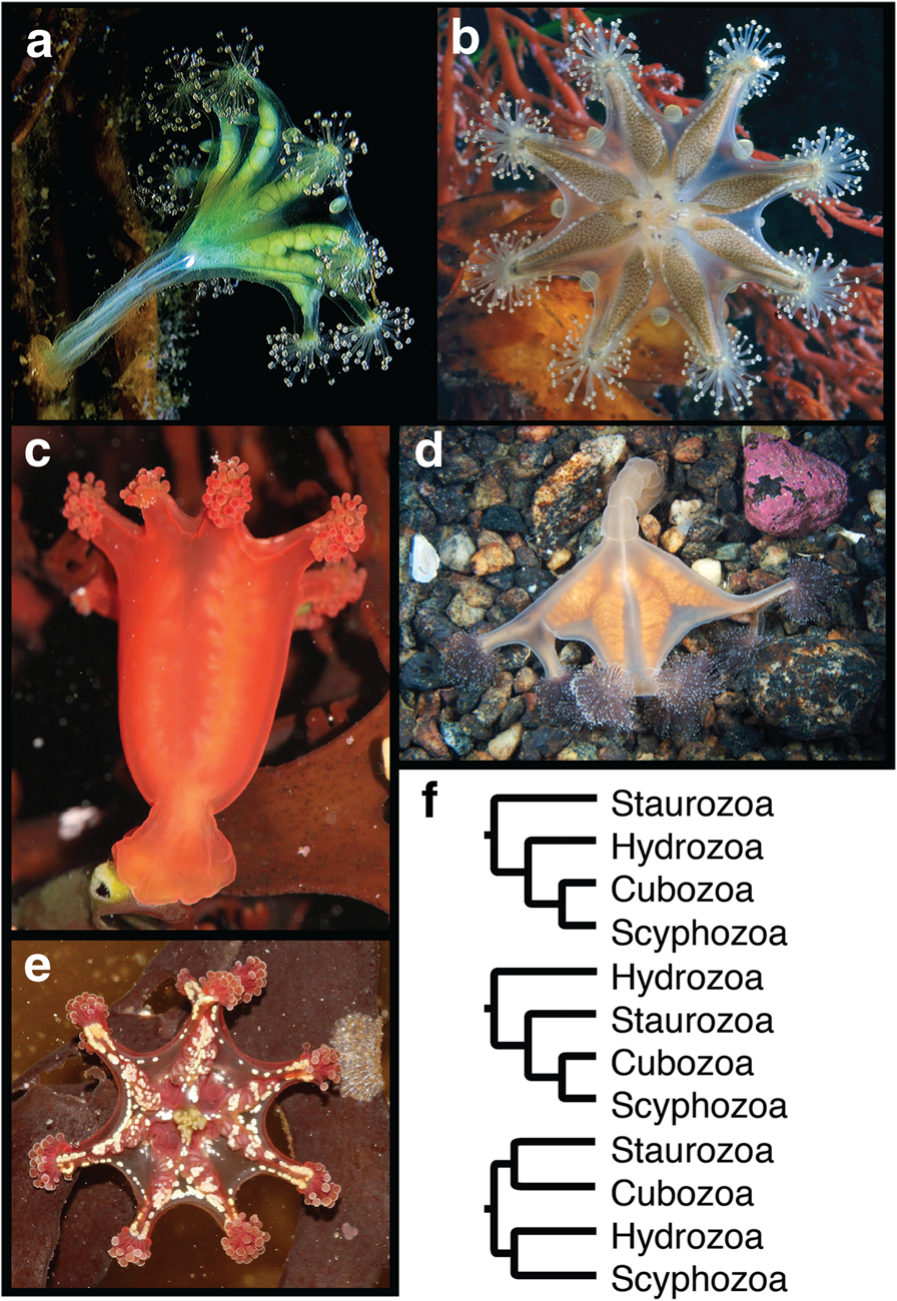
Exemplar staurozoans and competing phylogenetic hypotheses for the position of Stuarozoa within Medusozoa. **a** *Haliclystus auricula*. Credit: Marco Faasse; cropped. **b** *Haliclystus* “sanjuanensis”. Credit: Ron J. Larson. **c** *Craterolophus convolvulus*. Credit: Stauromedusae UK & David Fenwick; cropped. **d** Lucernaria quadricornis. Credit: Alexander Semenov. **e** *Calvadosia cruxmelitensis* Credit: Stauromedusae UK & David Fenwick; cropped. **f** Competing hypotheses for the phylogenetic position of Staurozoa within Medusozoa. Top from [25, 4] and Van Iten et al. [24]; bottom from Zapata et al. [10]

**Fig. 2:**
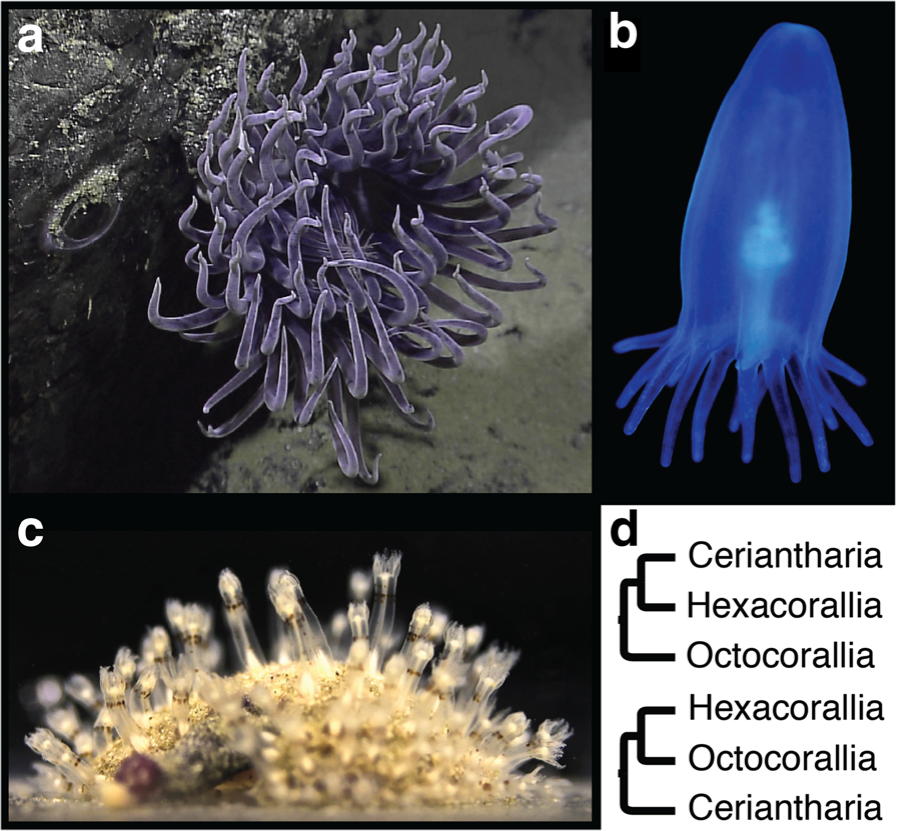
Exemplar ceriantharians and octocorals and competing hypotheses for the position of Ceriantharia within Anthozoa. **a** Ceriantharian tube anemone adult. Credit: NOAA Okeanos Explorer Program, Galapagos Rift Expedition, 2011; cropped. **b** Ceriantharian tube anemone pelagic larva. Credit: A. G. Collins, Bonaire, 2016. **c** Octocorallian, the sea pansy, *Renilla reniformis*. Credit: J. Ryan. **d** Competing hypotheses for the phylogenetic position of Ceriantharia within Anthozoa. Top from Daly et al. [31, 32] and Zapata et al. [10]; bottom from Stampar et al. [6]

Cnidarian phylogeny has been a source of debate for decades, with nearly every possible sister group relationship proposed among the major lineages of Medusozoa (Fig. 1) and Anthozoa (Fig. 2) [1, 4, 5, 6]. Whole mitochondrial phylogenomic analyses have supported paraphyletic Anthozoa and Scyphozoa [7, 8], but subsequent work suggested that these findings resulted from saturation bias [9]. More recent phylogenomic studies have supported the monophyly of Anthozoa and Scyphozoa [10] and placed Endocnidozoa as the sister group to Medusozoa [11]. However, these phylogenomic studies lacked several key taxa. For instance, Chang et al. [11] did not include data from Staurozoa, Ceriantharia, or Coronatae (Scyphozoa), while Zapata et al. [10] lacked data from Endocnidozoa and Rhizostomeae (Scyphozoa). In addition, data representation was sparse for Ceriantharia and Staurozoa in Zapata et al. [10] with weak support for the positions of both taxa represented by single exemplar species. Nevertheless, the topologies from these two independent phylogenomic studies were otherwise largely congruent, providing some prospect that large datasets and increased taxon sampling may settle long-standing questions about the evolutionary history of Cnidaria.

Staurozoa (Fig. 1) is one of the most poorly understood cnidarian clades. These animals have unique life history attributes, including non-ciliated, creeping, larval planulae that develop into polyps, which later undergo metamorphosis (unique from strobilation or budding) into adult stauromedusae [12, 13, 14]. In addition, the adult staurozoan body plan includes features common to both the polyp and medusa stages of other cnidarians, thus adding to their phylogenetic significance [15, 16, 17, 18]. The phylogenetic position of staurozoans relative to other medusozoan lineages remains controversial and recent hypotheses [7, 19, 10] present different implications for the evolution of medusozoan body plans (Fig. 1f). Analyses of rDNA, life history and anatomical features have suggested that Staurozoa is the sister lineage to the remainder of Medusozoa (Fig. 1f), [15, 20]. However, recent phylogenomic analyses [10] placed Staurozoa in a clade with Cubozoa and Scyphozoa (Fig. 1f middle). Support for the placement of Staurozoa has been characteristically low in these prior studies, leaving open the question of their phylogenetic position within Cnidaria. Critically, prior to the present study, relatively little evidence has been brought to bear on the phylogenetic position of Staurozoa, whether it be from morphology [21, 22, 23, 20, 24], a small number of genes [15, 25], or sparse representation (in terms of both data and taxon sampling) in an otherwise, large phylogenomic study [10].

Ceriantharia is another cnidarian clade whose uncertain phylogenetic placement has major evolutionary implications (Fig. 2). Ceriantharians are solitary tube-dwelling polyps with larval and adult stages that are morphologically distinct from the other anthozoan lineages (i.e., Octocorallia and Hexacorallia) [5, 6]. Ceriantharians differ from other anthozoans in that they possess a distinct planktonic larval stage known as the cerinula [26], a secreted composite tube into which they retract when disturbed, a novel type of structural cnidocyte called a ptychocyte that provides support for their tube dwellings [27] and two whorls of tentacles that surround the oral opening. Ceriantharia has been placed in several conflicting phylogenetic positions [28, 29] including in a recent study [6] as the sister lineage to the two main anthozoan lineages Hexacorallia and Octocorallia (Fig. 2f bottom). More commonly, Ceriantharia has been recovered as the sister to Hexacorallia, with which they share spirocytes, a common cnidocyte type absent from octocorals (Fig. 2f top) [30, 31, 32, 33, 7, 10]. As with Staurozoa, all previous analyses of the phylogenetic position of Ceriantharia have been based on morphology or limited sequence data, and support for the position in which Ceriantharia is recovered has been consistently low [6, 10].

There is also uncertainty surrounding the relationships of the major lineages within Hexacorallia. In several ribosomal and mitochondrial gene phylogenies, Actinaria (sea anemones) is recovered as the sister group to the remaining Hexacorallia [30, 34, 31, 32, 35]. However, in a more recent mitogenomic study, Zoantharia was recovered as the sister lineage to the remaining Hexacorallia [7]. Prior to the publication of molecular phylogenetic analyses, this latter relationship had been predicted based on morphological traits (e.g., mesentery arrangement). The recent publication of a study focused on toxin-related transcripts [36] allowed us to incorporate zoantharian transcriptomic data here, for the first time, in a phylogenomic study.

Phylogenomic analyses of genome-scale datasets (i.e., whole-genome-derived gene models or RNA-seq-derived transcripts) have recently been exploited to resolve a host of longstanding phylogenetic issues [37, 38, 39]. A critical step common to these analyses is the identification of one-to-one orthologs from genome-scale datasets for each taxon, which are then used as data partitions in large super-matrices. Various methods are available for the identification of such data partitions and methodological differences among them have been shown to impact phylogenetic inference [40, 41, 42]. Yet, phylogenomic analyses frequently rely on a single method for data matrix construction and do not examine the impact of alternative approaches on phylogenetic reconstruction (e.g. [37, 43, 44]).

Here, we apply new phylogenomic data for Staurozoa, Ceriantharia and several other previously under-sampled cnidarian clades to the construction and analyses of independent phylogenomic datasets for Cnidaria using two popular approaches: *1)* Agalma [45] and *2)* a custom pipeline based on Orthofinder [46] and PhyloTreePruner [47]. We show that both procedures produce datasets with surprisingly little overlap in terms of data composition, but resolve the same topology under robust phylogenetic methods. We then leverage our highly resolved cnidarian phylogeny to address questions surrounding the origins and evolutionary histories of several key organismal innovations in Cnidaria. Our character mapping studies, based on explicit statistical models, identify key evolutionary transitions within Cnidaria and suggest that the ancestral cnidarian was a solitary polyp that lacked a medusa stage or a photosynthetic endosymbiont. Further, our analyses strongly suggest that symbiosis with photosynthetic eukaryotes has evolved on multiple occasions in Cnidaria.

## Results

We generated transcriptomic data from five staurozoans (*Calvadosia cruxmelitensis*, *Craterolophus convolvulus*, *Haliclystus auricula*, *Haliclystus “sanjuanensis”* and *Lucernaria quadricornis*), one scyphozoan *Cassiopea xamachana* and the cerianthid *Cerianthus borealis*. In addition, we sequenced and generated a rough-draft assembly of the nuclear genome of *Renilla reniformis*. The genome assembly had an N50 of 1843 base pairs. We predicted 12,689 protein-coding genes, many of which are likely partial, but sufficient for downstream phylogenomic analyses. We also used the highest quality transcriptomic data from Zapata et al. [10], to which we added genomic and transcriptomic data from several taxa that were underrepresented in previous studies, including most endocnidozoan taxa from Chang et al. [11]. After an initial round of matrix construction and phylogenetic analyses, several new cnidarian transcriptome datasets became available, and we incorporated an additional 13 taxa into our final data matrix OF-PTP_75tx (Additional files 1 and 2).

### Potential contamination identified in cnidarian transcriptome data

**Fig. 3:**
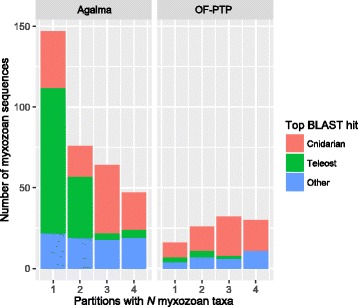
Relationship between sparse data representation and the retention of contaminated sequences in phylogenomic data matrices as illustrated by myxozoan species. We conducted BLAST similarity searches against a metazoan genome database for all myxozoan sequences present in both the AG_62tx and OF-PTP_62tx matrices. In addition, we noted how many myxozoan species were present in each partition. Myxozoans are internal parasites of teleost fishes and we noted significant contamination in transcriptome data from these host species. The Agalma pipeline produces a large, but sparse matrix as compared to OF-PTP (Fig. 4). In cases where contamination is common, as with myxozoan data, sparse data matrices have high numbers of partitions with single species represented per clade, which in turn are enriched for contaminant sequences. Partitions with greater than one species of myxozoan present have a lower potential to include contamination. The OF-PTP pipeline produces a denser data matrix, which makes it inherently less prone to selecting contaminants

We applied a strict filter to all datasets to remove potential contaminants. In total, we removed less than 5% of sequences from most datasets except for the following taxa: *Alatina alata* (7.9%), *Anemonia viridis* (6%), *Anthopleura elegantissima* (7%), *Gorgonia ventalina* (6.8%), *Hydractinia polyclina* (6.8%), *Platygyra carnosus* (6.7%), and *Seriatopora hystrix* (6.9%). In addition, many sequences from the myxozoans *Kudoa iwatai* (39.8%), *Myxobolus cerebralis* (25.6%), *M. pendula* (40.5%), and *Thelohanellus kitauei* (21.4%), as well as the filiferan hydrozoan *Podocoryna carnea* (26.7%) had best matches to bilaterian sequences and were subsequently removed (Additional file 1). Following the removal of these putative contaminants, preliminary phylogenetic analyses showed that the myxozoan sequences procured by the Agalma pipeline still retained many contaminants, as these species were positioned within the vertebrates (Additional file 3). Further analysis of myxozoan-bearing partitions from both datasets showed that the Agalma pipeline was prone to include partitions with a single myxozoan species present and that these partitions were more likely to be comprised of contaminants, a situation not encountered in the OF-PTP dataset (Fig. 3). Further, when Agalma partitions with greater than three myxozoan species were selected for phylogenetic analysis (47 partitions), the myxozoan species were resolved in their expected position within the Endocnidozoa, and the remaining topology was largely consistent with all other results, see below (Additional file 4).

### Phylogenomic matrix generation pipelines produced contrasting data matrices

**Fig. 4:**
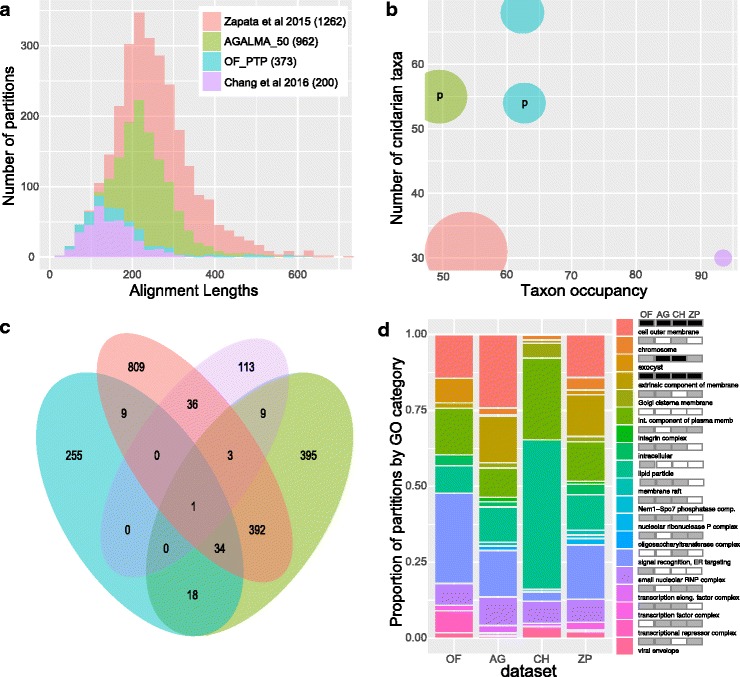
Comparisons of new and previously published phylogenomic datasets for Cnidaria reveal significant differences. **a** Histograms showing the distributions of alignment lengths for partitions included in the OF-PTP_62tx, AG_62tx and two previously published phylogenomic matrices [11, 10]. **b** The number of taxa plotted against the average taxon occupancy for each dataset. Size of each circle is based on number of partitions (see inset in **a**). p denotes preliminary datasets. **c** Venn diagram indicating the overlap in *N. vectensis* gene identities from partitions present in each dataset. The most similar datasets are AG_62tx and that from Zapata et al. [10], which are both based on Agalma [45]. **d** Composition, enrichment and depletion of GO terms associated with the cellular component category from the *N. vectensis* sequences present across partitions. Left, the composition of unique cellular component terms are shown as bar plots for each dataset. Only terms that are significantly enriched or depleted relative to their frequencies in the *N. vectensis* protein set in at least one dataset are shown. Right, the identities of each cellular component term and their enrichment or depletion for each dataset. Black = depleted. White = enriched. Grey = not significant. For **b**-**c**, datasets are color coded as in **a**

We built two preliminary, independent phylogenomic data matrices with Agalma (AG_62tx) and OF-PTP (OF-PTP_62tx). After selecting orthologous partition alignments that exceeded 50% taxon occupancy, the Agalma pipeline incorporated roughly three times as many genes and four times as much data (962 single-gene partitions, 233,568 data positions) as OF-PTP (372 single-gene partitions, 53,389 data positions) (Fig. 4). Furthermore, the average partition length was longer for the AG_62tx dataset than OF-PTP_62tx (Fig. 4a). Comparisons of the across-partition *N. vectensis* complements of AG_62tx and OF-PTP_62tx revealed that only 53 loci are shared between the two datasets (Fig. 4c).

We also detected substantial differences in taxon occupancy between the datasets produced by Agalma and OF-PTP. AG_62tx had, on average, significantly lower taxon occupancy (481/962 = 49%) compared to OF-PTP_62tx (232/372 = 62%; Fisher’s Exact Test *P* = 0.028) or OF-PTP75tx (225/357 = 63%; Fisher’s Exact Test *P* = 0.023). In addition, the AG_62tx dataset had exceedingly low coverage for several key groups (see below and Additional files 3, 5 and 6). Similarly, the OF-PTP datasets were substantially denser, but smaller in size (Fig. 4a-b) than the dataset reported by Zapata et al. [10], which was also constructed using the same Agalma pipeline [45] (Fig. 4b, Additional files 3, 5 and 6). The data matrix reported by Chang et al. [11], a manually curated dataset, had the highest density, but the lowest number of ingroup taxa present (Fig. 4b). Overall, our findings suggested significant differences between the composition of datasets produced by OF-PTP, Agalma and those reported in recent phylogenomic analyses of Cnidaria [11, 10].

Next, we sought to understand how these datasets differed in terms of the functional classes of genes present in each. We first compared the number of *N. vectensis* genes shared in each dataset and found surprisingly low levels of overlap among datasets (Fig. 4c). This estimation could be confounded if different pipelines retained different, but closely related, *N. vectensis* paralogs during their distinct tree pruning procedures, potentially exaggerating differences between them. We therefore conducted gene ontology (GO) analyses of enrichment and depletion by comparing the relative proportions of each GO term for the *N. vectensis* genes present in each dataset to their relative proportion in the background *N. vectensis* v1.0 protein set [48]. These analyses show that significant differences in GO term representation, relative to the background, pervade each dataset across each GO category examined (e.g. cellular component, molecular function and biological process). These differences are evident by comparing the composition and relative enrichment and depletion of GO terms between each of the data matrices (Fig. 4d, Additional file 7). Our findings demonstrate that the two independent datasets produced here, together with those from the two most recent phylogenomic analyses of Cnidaria [11, 10], are comprised of data partitions that differ profoundly in terms of gene identity and functional class (Fig. 4c-d).

### Consistent phylogenomic results from different data matrices

**Fig. 5:**
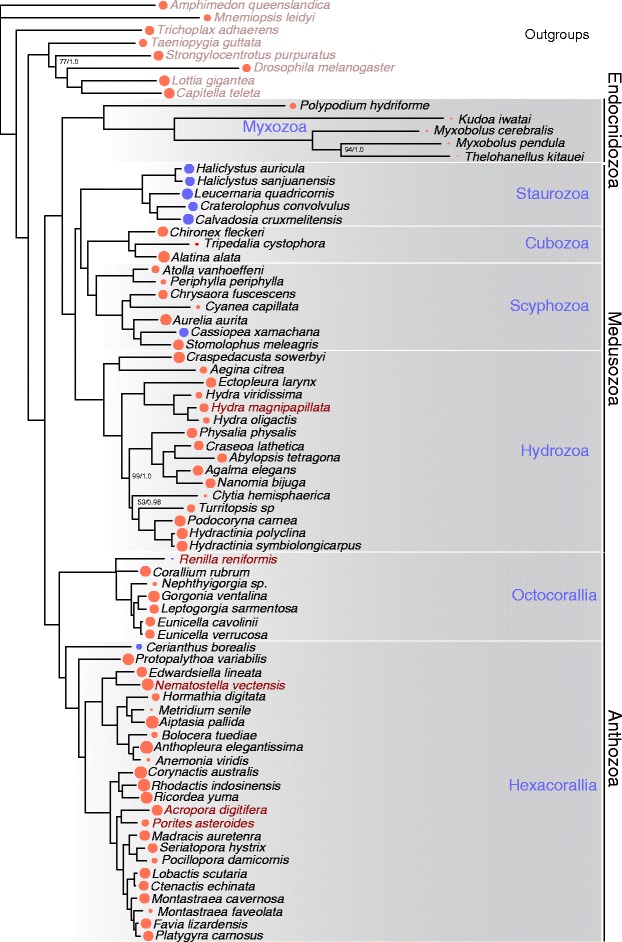
ML and Bayesian analyses of the OF-PTP_75tx dataset confidently resolve cnidarian phylogeny and depict the three major lineages. Shown is the topology from ML analyses (Additional file 9). Nodes not present in the Bayesian analysis (Additional file 10) are collapsed to polytomies. All nodes receive maximum support in both analyses except where indicated as bootstraps and posterior probabilities. Circles at terminal tips indicate the number of data partitions present per taxon. Blue circles indicate data produced here, while orange circles indicate publically available data. All datasets are derived from RNA-seq data except when whole genome assemblies where used as indicated in Red

Preliminary phylogenetic analyses of the AG_62tx and OF-PTP_62tx matrices were largely congruent in our ML analyses, with the exception that the Myxozoa was unexpectedly positioned within the vertebrates in our analyses of AG_62tx (see above; Additional file 3). The unexpected position of Myxozoa in the AG_62tx analysis is likely due to contamination exaggerated by data sparseness in that matrix. On average, myxozoan species are represented in a significantly smaller proportion (Fisher’s Exact Test) of partitions in the AG_62tx dataset as compared to the OF-PTP_62tx dataset: *Myxobolus pendula P* = 0.027; *Thelohanellus kitauei P* = 0.0001; *Myxobolus cerebralis P* = 0.0001 (Fig. 5, Additional file 3). We hypothesized that if contaminants were still present in the myxozoan datasets despite our filtering efforts, they would be minimized in partitions that had more than one myxozoan species present, as the likelihood of selecting more than one orthologous contaminant sequences from multiple datasets for the same partition would be low. In fact, we recovered a monophyletic Cnidaria with the AG_62x matrix after pruning myxozoan partitions with fewer than three myxozoan species (Additional file 4). The sparse representation of myxozoan sequences across AG_62tx makes it more likely that a single myxozoan species is represented per partition, as compared to the less sparse OF-PTP matrix (Fig. 3). This relationship, in turn, makes it more likely that contaminants are incorporated into the Agalma matrix. Given the sparse nature of the matrix produced by Agalma under default settings, we decided to focus our deeper analyses on the matrix derived from the OF-PTP approach.

After our initial analyses, we added new data for 13 additional taxa derived from our own sequencing efforts and from newly published studies, for a total of 67 cnidarian taxa (75 species in total, Additional files 1 and 2) and produced a new dataset containing 357 partitions (53,539 positions) which we refer to as OF-PTP_75tx (Additional file 8). We performed both ML and Bayesian analyses on OF-PTP_75tx (Fig. 5, Additional files 9 and 10). After pruning *T. adherens* from the trees sampled from both chains of the PhyloBayes run, we obtained a convergence with high confidence (maxdiff = 0.152284). In addition, *Renilla reniformis*, which had among the most limited data representation of any taxon, formed a monophyletic group with *Corallium rubrum* in the Bayesian analyses, but was the sister to the remaining octocorals in ML analyses. We therefore collapsed the two conflicting nodes into polytomies (Fig. 5, Additional files 9 and 10). Overall, results from Bayesian and ML analyses were congruent, with all but three nodes in the cnidarian ingroup receiving maximum support in both analyses.

The following phylogenetic findings related to our analyses of OF-PTP_75tx are recovered in both ML and Bayesian analyses and receive maximum support in each as detailed in Fig. 5. We recovered a monophyletic Anthozoa as sister to a clade containing Medusozoa plus Endocnidozoa. In addition, Ceriantharia, represented by *Cerianthus borealis*, is sister to Hexacorallia. Within Hexacorallia, we confirmed many previous studies that recovered Scleractinia and Corallimorpharia as sister taxa (e.g. [31, 32, 49, 50]), but unexpectedly recovered Zoantharia as the sister lineage to the remainder of Hexacorallia. Consistent with Chang et al. [11], our analyses recovered a monophyletic Endocnidozoa (Myxozoa + Polypodiozoa) as sister to Medusozoa. Our analyses split Medusozoa into two monophyletic groups consisting of Hydrozoa (comprised of monophyletic Hydroidolina and Trachylinae; [51, 52, 1], ) and Acraspeda, a lesser-known clade uniting Staurozoa, Cubozoa and Scyphozoa. Within Scyphozoa, we recovered a paraphyletic Semaeostomeae where *Aurelia aurita* grouped with Rhizostomeae. Within Hydrozoa, Trachylinae is the sister lineage to the remaining Hydroidolinia, which is further divided into Aplanulata and a clade comprised of siphonophores, the leptothecate *Clytia hemisphaerica* and species of the Filifera IV group.

### The histories of key cnidarian traits

**Fig. 6:**
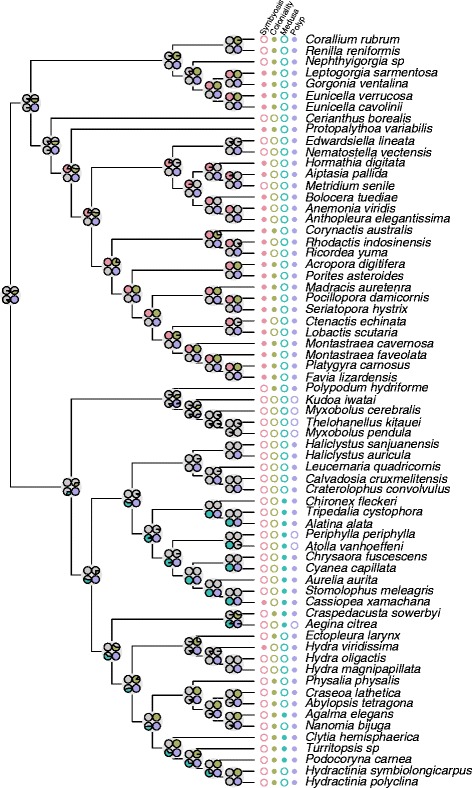
Studies of character evolution reveal a dynamic history of gain and loss for each trait examined. We conducted stochastic character mapping [53, 55] on our cnidarian ingroup topology (branch lengths not shown) for each character state included. Ancestral state reconstructions of each character at each node are shown as pie charts representing posterior probabilities. The color-coded presence or absence of each character is shown at Right. Our results suggest that the ancestral state of Cnidaria was a non-symbiotic, solitary polyp, however, other characters are equivocal at this node

We applied stochastic character mapping [53, 54, 55] to reconstruct ancestral character states for selected traits on our topology (Fig. 6). In addition, we conducted a Bayes Factor test comparing the prior and posterior probabilities of each trait evolving either once or multiple times [56] using a range of gain and loss rate parameters including empirical estimates [57] (Table 1). These analyses provide complementary views of character evolution. Of the characters we examined, we recovered strong support for multiple origins of the intracellular, autotrophic, eukaryotic symbiont character (*P* = 0.96; Table 1), which occurred independently within all major classes of Cnidaria except the parasitic Endocnidozoa (Fig. 6). In contrast, results from our analyses of coloniality were less clear. We found marginal support for a single origin of coloniality across the tree (Table 1; *P* = 0.83) while ancestral state reconstructions also provided marginal support for the hypothesis that the last common ancestor of the included cnidarian taxa possessed the alternative, solitary, character state (PP = 0.76).

**Table 1:** Bayes factor analyses of single vs. multiple origins of selected traits

Trait	Priors on rates of gain:loss	BF of single (HO) vs. multiple origins (HA)	Log10(BF)	2xlog_e(BF)	Posterior Probability (HO)	Posterior Probability (HA)
Symbiosis	0.47:0.47 ^a^	0.0368	−1.4338	−6.6028	0.0355	*0.9645*
	10:1	0.0086	−2.0675	−9.5214	0.0085	*0.9915*
	1:10	0.0004	−3.3976	−15.6467	0.0004	*0.9996*
Coloniality	0.7:0.7 ^a^	5.0038	0.6993	3.2204	0.8334	0.1666
	10:1	1.5281	0.1841	0.8480	0.6044	0.3956
	1:10	1.5601	0.1932	0.8896	0.6094	0.3906
Medusa	0.27:0.27 ^a^	63.4250	1.8023	8.2998	*0.9845*	0.0155
	10:1	4.3807	0.6415	2.9544	0.8142	0.1858
	1:10	3.1507	0.4984	2.2952	0.7591	0.2409
Polyp	0.2:0.2 ^a^	3.5403	0.5490	2.5284	0.7797	0.2203
	10:1	12.9087	1.1109	5.1158	0.9281	0.0719
	1:10	8.4292	0.9258	4.2634	0.8939	0.1061

Values in italics have posterior probabilities greater than 0.95

*HO* null hypothesis that trait originated once

*HA* alternative hypothesis that trait evolved more than once

^a^gain/loss rates estimated from observed data using *corHMM* [4]

Life history stages within Cnidaria are strikingly plastic, making universal definitions difficult [108, 59, 23, 60, 3]. Here, we consider the medusa to be a sexually mature, solitary, free-swimming life history stage that spawns after separation or metamorphosis from a polyp. We consider the polyp stage to be a post-larval non-medusa stage. Our ancestral state reconstructions recovered only equivocal support (PP = 0.52) for the presence of medusa in the last common ancestor of Medusozoa with several inferred losses throughout the group, most likely in the lineages leading to Staurozoa and again within Aplanulata and Siphonophora. However, we recover strong support for a single origin of medusae (*P* = 0.98) on our tree (Table 1). The polyp life history stage is common across the cnidarian phylogeny with notable losses in Endocnidozoa and Trachylinae. A single origin of the polyp stage is well supported (*P* = 0.93) and the last common ancestor of our well-sampled cnidarian phylogeny is strongly inferred to have a polyp stage (PP = 1.0). This finding is consistent with the conventional view of cnidarian body plan evolution [5, 61] and recent fossil evidence form the lower Cambrian [62]. Our results from Bayes Factor tests for multiple origins are based on empirically derived gain and loss rate parameters, but are robust to a wide range of exaggerated rates of gain and loss (Table 1).

## Discussion

### The phylogenetic structure of Cnidaria

**Fig. 7:**
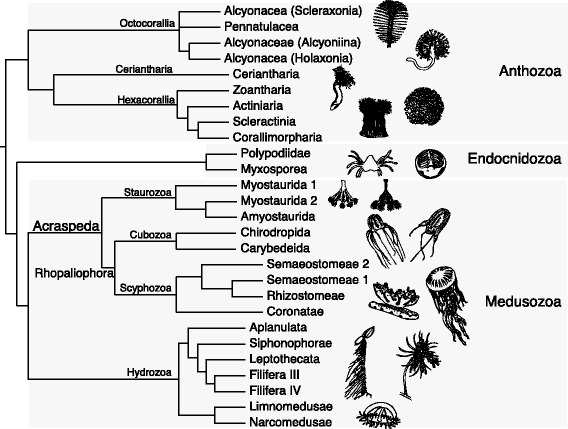
Summary of results. Our working hypothesis for the topology of major cnidarian lineages based on the present study

Cnidaria is a large and diverse clade that has produced numerous fascinating evolutionary novelties since at least the Cambrian [51, 62]. Understanding the origin and evolution of these innovations requires a stable phylogenetic framework, but resolving the relationships of the major cnidarian lineages has eluded evolutionary biologists. Here we present a well-resolved cnidarian phylogeny using genomic and transcriptomic data from representatives of all classes, summarized in Fig. 7.

Our analyses support the monophyly of Anthozoa, with the enigmatic Ceriantharia placed as sister to Hexacorallia. Surprisingly, we recover Zoantharia as the sister group to the remaining Hexacorallia, whereas most other molecular phylogenetic studies placed Actinaria in this position (e.g. [31, 32]). This placement of Zoantharia has several implications for morphological evolution within Hexacorallia. For example, Zoantharia, like Ceriantharia and Octorallia, but unlike most other Hexacorallia, have a single siphonoglyph (a ciliated groove located in the actinopharynx) and are therefore bilaterally symmetrical [63]. Thus, our placement of Zoantharia as sister to the remaining Hexacorallia adds support to the idea that the last common ancestor of Anthozoa (and perhaps Cnidaria) was bilaterally symmetrical. We were unable to include Antipatharia in our dataset and data from this group will be important in future phylogenomic studies for solidifying the hexacorallian topology.

Our analyses corroborate previous findings of a sister relationship between Medusozoa and Endocnidozoa (Myxozoa and *Polypodium hydriforme*) [11, 64, 65]. Importantly, our findings strongly support the existence of a clade consisting of Staurozoa, Cubozoa and Scyphozoa, which received only weak support in previous analyses [10]. Our placement of Staurozoa revives evolutionary hypotheses put forward more than half a century ago by Hyman [21] and Thiel [66] and includes the union of the rhopalia-bearing Cubozoa and Scyphozoa in the clade Rhopaliophora [67].

Our study provides the most comprehensive taxon sampling of Cnidaria for phylogenomic analysis to date (Additional file 11). While our attempts to account for potential contamination resulted in the dramatic reduction of data for some taxa, we managed to recover sufficient partitions with at least 50% taxon occupancy to estimate a robust phylogeny for Cnidaria (Fig. 5). We show that given the same input data, the Agalma pipeline under default parameters [45] tended to produce larger though sparser data matrices than the OF-PTP procedure [46, 47]. In our analyses, the data matrix produced by the Agalma pipeline resulted in the misplacement of the data-poor Myxozoa within vertebrates due to contamination (Fig. 3, Additional files 3 and 4) despite our extensive data filtering steps. We note that our analyses are based on the earlier version of Agalama v0.5-devel and a subsequent update has been made available (https://bitbucket.org/caseywdunn/agalma).

The minuscule amount of overlap between the Agalma and OF-PTP datasets (only 53 *N. vectensis* loci shared across partitions between AG_62tx and OF_62tx; Fig. 4c) was surprising, given that both pipelines use similar approaches: TransDecoder [68] to produce translated peptidomes, all-by-all BLAST to generate similarity graphs and Markov clustering (MCL; [69]) to define orthologous gene clusters. Why would similar approaches produce largely different datasets using identical input data? Minor differences between the Agalma and OF-PTP pipelines include the default MCL inflation parameters (2.1 and 1.5 respectively). However, it has been demonstrated that varying the inflation parameter in MCL clustering does not have a major effect on resulting orthogroups (Li et al. [70]; but see Gibbons et al. [71]). One important difference that could explain the construction of largely different datasets by the two pipelines is the procedure used for pruning orthogroups when multiple representative sequences from each taxon are present. The treeprune procedure in Agalma may produce several partitions per orthogroup, while the OF-PTP pipeline uses PhyloTreePruner [47], which produces only a single partition (the largest monophyletic group) from each orthogroup, regardless of the topology. Differences in orthogroup pruning are also likely to drive differences in matrix sparseness, which can exacerbate the influence of contamination in phylogenomic datasets (Fig. 3). While there are advantages and limitations to each approach, the important consideration here is that phylogenetic analyses of very different matrices produced by both pipelines yielded identical topologies when free of contamination. (Additional file 3).

### Deciphering relationships within Anthozoa

This study is the first to confidently determine the position of the ceriantharian tube anemones (Fig. 2) as the sister group to Hexacorallia within Anthozoa (Figs. 5 and 7). Our result contradicts the favored hypothesis of Stampar et al. [6] that Ceriantharia is the sister to the remaining Anthozoa, and corroborates earlier but weakly supported hypotheses based on morphology [31, 32], mitochondrial genomes [7] and phylogenomic datasets [10]. Ceriantharians possess several unique characteristics [31, 32] and following the suggestion of Stampar et al. [6] we treat it as a unique taxon among the other anthozoan clades (Fig. 7). Although often treated as members of Hexacorallia [31, 32], ceriantharian mesenteries, which divide the gastric cavity, are coupled, but not paired as they are in Hexacorallians [72]. Both taxa do, however, possess a distinctive nematocyst type known as a spirocyst, which is likely a synapomorphy for the clade consisting of Ceriantharia plus Hexacorallia [31, 32].

In addition to other unusual characteristics, ceriantharians possess swimming larvae called cerinula that are somewhat similar to medusae (Fig. 2b) [26]. In some cases, these stages even develop gonads [73, 74, 75] and indeed such stages are responsible for one of the more interesting, if obscure, confusions in cnidarian biodiversity studies. Haeckel [76] observed several such stages and erected a family, Tesseridae, that he concluded was part of Stauromedusaea, whose members are otherwise benthic (see below). For decades, the swimming pelagic species of Tesseridae went mostly unobserved and were neglected in compendia of known medusae [77] until Goy [78] reported an observation and documented its veracity. It was not until a few years ago [79] that Goy’s species and Haeckel’s family were recognized for what they are, precocious larval tube anemones that had yet to settle and secrete their tubes into which they would project their soft adult bodies.

Our taxon sampling for Anthozoa is sufficient to provide confidence in the relationships of the major hexacorallian lineages including the position of Ceriantharia as the sister group to Hexacorallia (Figs. 5 and 7). Within Hexacorallia, it will be important for future studies to incorporate species from Antipatharia (black corals) to more fully understand the topology of this group. Likewise, several open questions regarding relationships within Octocorallia remain [80, 81]. The majority of our sampling is limited to Holaxonia, a suborder of Alcyonacea. Addressing these issues will require increasing the breadth of taxon sampling across Alcyonacea (e.g. Calcaxonia, Protoalcyonaria, Scleraxonia, Stolinofera) and the inclusion of Helioporacea (e.g., the reef-forming blue corals) in future phylogenomic studies.

### Resolving key controversies within Medusozoa

The benthic Stauromedusae of the class Staurozoa have had a long and confused taxonomic history (Fig. 1). The earliest studies classified them as anthozoans in the nineteenth century taxon Polypi of the Actiniae [82, 83, 84], but Sars [85] was the first to note that the finger-like gastric cirri and the four-part arrangement of gonads in Stauromedusae bore a striking resemblance to similar features of non-hydrozoan medusae. Indeed, our results strongly suggest that gastric cirri and a quadripartite body plan are synapomorphies of the clade uniting Staurozoa, Cubozoa and Scyphozoa, although the presence of four sets of longitudinal muscles in some endocnidozoans (e.g., malacosporean myxozoans) could suggest that a quadripartite body plan is a plesiomorphy for Medusozoa that was lost in Hydrozoa and other endocnidozoans [86]. In the late 1800’s, the so-called stalked jellyfishes were the subject of discourse in the nascent field of evolutionary biology where they were viewed as “degenerate scyphomedusae” [87], “arrested scyphistoma[e]” [88], or as “ancestral forms” representing an early diverging lineage “equivalent in value” to the scyphomedusae [89]. This earlier view is borne out by our results showing strong support for Staurozoa as the sister group to Cubozoa plus Scyphozoa (Figs. 5 and 7) and is also in agreement with the topology of Zapata et al. [10], which had only weak support. Earlier phylogenetic analyses of rDNA and morphology also supported the view that Staurozoa is a distinct clade from Scyphozoa and Cubozoa, but instead suggested that Staurozoa was the sister group to the remaining medusozoans [4, 24].

Our data strongly suggest that Staurozoa is a member of a monophyletic group containing Cubozoa and Scyphozoa. The earliest taxon name that could apply to this clade is Acraspeda [90], which was originally restricted to scyphozoan and cubozoan species, but later included Staurozoa in a discussion of an evolutionary series linking Stauromedusae to Coronatae and Discomedusae [91, 76, 92, 93]. During this same period, Goette [94] originated the name Scyphozoa and included Stauromedusae as one of its orders. Based on distinct life cycle and polyp traits, Werner [95] extracted Cubomedusae from Scyphozoa as the Cubozoa, and by a similar analysis of life history and anatomical traits, Marques and Collins [23] established Stauromedusae as the medusozoan class Staurozoa. In addition, the first explicit name for Cubozoa plus Scyphozoa, the Rhopaliophora, was introduced by Ax [67] and we follow that here, while using Gegenbaur’s Acraspeda as the clade uniting Staurozoa and Rhopaliophora (Fig. 7). We note that Haeckel [76] appears to be the first to use the clade name Acraspeda in its present sense.

### Evolution of complex characters in Cnidaria

Our phylogenomic analyses of Cnidaria provide a framework for understanding the evolutionary histories of several important organismal traits that likely contributed to the success of the phylum. Our inclusion of all major lineages makes this phylogenetic hypothesis particularly suitable for reconstructing ancestral states for the last common ancestor of Cnidaria. We scored each taxon in our dataset for the presence or absence of recognizable traits including photosynthetic eukaryotic endosymbionts, colonial body plans, a medusa stage and a polyp stage as discrete characters (Fig. 6). We used explicit models of character evolution to reconstruct individual character histories [53, 54, 55], rates of gain and loss [57] and numbers of origins [56]. These methods provide powerful tools for understanding the evolutionary histories of selected traits, but several caveats regarding their application are in order. First, the efficacy of ancestral state reconstruction is dependent upon taxon sampling and, while our dataset is larger than previous phylogenomic analyses of cnidarians (Fig. 4b), we emphasize that taxon sampling in certain octocoral and hydrozoan subclades remains sparse and characters that vary within orders may be obscured. In addition, our taxon selection likely over-represents nearshore, shallow-water taxa, which could bias our attempts to reconstruct the ancestry of traits like symbiosis or even life history. Finally, the statistical approaches employed here depend on parameters that define the rates of character transitions. For ancestral state reconstructions, a one-rate model was a significantly better fit for each character (Table 1) and so we choose to use equal rates on gain and loss. For studies of independent origins of traits we chose to conduct a sensitivity analysis that included a wide range of exaggerated rate priors, in addition to an empirically estimated rate prior. In this case, our results were robust to such parameter differences, even when exaggerated (Table 1).

The acquisition of phototrophic endosymbionts, including *Symbiodinium* and/or zoochlorellae, constitutes a major ecological innovation in the evolutionary history of Cnidaria allowing these organisms to thrive in oligotrophic waters [96]. Endosymbiosis in hexacorals, especially the scleractinian corals, is a major area of interest considering the sensitivity of this symbiotic relationship in a changing environment, but is also found in every other cnidarian class except Endocnidozoa. Our analyses support the hypothesis that endosymbiosis has evolved multiple times during the evolutionary history of Cnidaria, with independent origins likely occurring in hexacorals (see also [97]), octocorals, scyphozoans and hydrozoans (Fig. 6). This finding may highlight both the adaptive utility of photosynthetic endosymbionts in oligotrophic environments and the possibility of a shared underlying mechanism, such as the ability to absorb dissolved nutrients across epithelial membranes, for the establishment and maintenance of endosymbiotic autotrophs across disparate cnidarian clades [3].

Colonial organization among disparate cnidarian clades may facilitate adaptations related to maximizing nutrient and spatial resources, defense, surface:volume constraints of unitary animals and reproductive success. Further, coloniality is associated with enhanced modularity and the potential for division of labor among zooids (somatically integrated individuals that arise by budding or division) [98]. Division of labor of this type reaches its pinnacle in the siphonophores where it parallels the level of functional specialization exhibited by the cells of other multicellular organisms [99]. Our analysis of the character history of coloniality across Cnidaria shows that this trait was likely present in the last common ancestor of octocorals, scleractinians and hydrozoans, but was absent or equivocal in all other deeper nodes including the last common ancestor of Cnidaria. We note that our finding is at odds with previous studies of scleractinians, which included much greater taxon sampling. These studies revealed a more dynamic evolutionary history for coloniality [97] and suggested that the last common ancestor of Scleractinia was solitary [100]. Strong evidence for loss of coloniality in our dataset is found only in the Aplanulata hydrozoans, which include species within the genus *Hydra*.

The polyp is generally regarded as the ancestral life history state in Cnidaria, to which the medusa was added in one or more lineages ([5, 61]). Defining different cnidarian life history stages is often difficult because of the many variations and exceptions exhibited within the wide variety of taxa [58, 59, 23]. Our definition of the medusa as a liberated, propulsive form bearing gonads [58] requires that the solitary, benthic members of Staurozoa are scored as possessing a derived polyp rather than a degenerated medusa as suggested earlier [4, 7, 66, 101, 95] and the stolon stage of *Polypodium hydriforme* is scored as neither a medusa nor a polyp [102].

Our analyses strongly support the polyp-first hypothesis [21, 95] with the prediction of a polypoid ancestor to Cnidaria, with at least two independent losses of the polyp stage in lineages leading to Endocnidozoa and within Trachylina [103] (Figs. 6 and 7). Previous studies have suggested a single innovation of the medusa form within Medusozoa [7], with independent losses in several Hydrozoa clades [58]. Our results also favor a single origin of medusa with independent losses of this stage in the lineages leading to Staurozoa and Aplanulata (Table 1, Fig. 6). These analyses illustrate the remarkable variation of life history strategies within Cnidaria and set the stage for research into the genomic and developmental factors underlying these transitions.

## Conclusions

Cnidaria have experienced more than 600 million years of independent evolution and in the process generated an array of biological innovations. Some of these innovations (e.g., cnidocytes) evolved in the stem of Cnidaria, but many of the most intriguing (e.g., endosymbiosis, coloniality and the medusa life history stage) likely evolved after the last common cnidarian ancestor and were lost in some lineages. The well-resolved phylogenetic relationships put forth in this study, as well as the ancestral reconstruction of some of these traits marks a major step toward understanding the extraordinary evolutionary history of Cnidaria. While our analyses do not reveal the states of all our selected characters with confidence in the ancestral cnidarian, a non-symbiotic, solitary polyp that lacked a medusa stage remains the most likely prediction, with multiple independent origins of symbiosis occurring subsequently.

## Methods

### Taxon sampling and sequencing

We generated new transcriptome data from a range of cnidarian taxa including five staurozoans (*Calvadosia cruxmelitensis*, *Craterolophus convolvulus*, *Haliclystus auricula, Halyclystus “sanjuanensis” (nomen nudum)* and *Leucernaria quadricornis*), one cerianthid (*Cerianthus borealis*), one scyphozoan (*Cassiopea xamachana*) and gene model data from whole-genome sequencing of one octocoral (*Renilla reniformis*). To these we added the following previously published data: 13 cnidarian transcriptomes from Zapata et al. [10], 30 RNA-seq datasets from the NCBI SRA Archive and 16 transcriptomes and gene models from whole-genome data. We included the same seven outgroups used by Zapata et al. [10] to which we added *Lottia gigantea* [104].

A single adult sample of *Calvadosia cruxmelitensis* was collected from Penzance, Cornwall, England. A single adult sample *Cerianthus borealis* was collected near Shoals Marine Laboratories, Appledore Island, Maine, USA. An adult sample of *Craterolophus convolvulus* was collected from Rye Harbor, Rye, New Hampshire, USA. An adult sample of *Haliclystus auricula* was collected from Eastport, Maine, USA. *Haliclystus “sanjuanensis”* samples of various sizes (juveniles and adults) were collected from Cattle Point, Friday Harbor, Washington, USA. An adult sample of *Lucernaria quadricornis* was collected near Shoals Marine Laboratories, Appledore Island, Maine, USA. *Cassiopea xamachana* samples were from a lab culture originally collected from Key Largo, Florida, USA. The *Cassiopea xamachana* transcriptome was generated from three clonal lines (T1-A, T1-B, T2-B) at four stages (aposymbiotic, 3 and 8 days post-inoculation by *Symbiodinium microadriaticum* and strobila stage). *Renilla reniformis* adult sample was collected in the surf at Fort George Inlet, Jacksonville, Florida, USA. Washington specimens were collected with permission from Friday Harbor Marine Labs. Florida specimens were collected within allowable limits as stipulated by Florida Fish and Wildlife Conservation Commission. New Hampshire collections were done under a permit from the New Hampshire Fish and Wildlife Department. Maine collections were done under a permit from the State of Maine Department of Marine Resources. England collections were done under a permit from Natural England.

Additional details on data sources are provided in Additional files 1 and 2. Materials used for sequencing were either sampled from whole organisms, or from multiple tissue types per taxon as to broaden transcript diversity. Further details, including extraction methods, DNA and RNA-seq library preparation and sequencing are provided in Additional file 12.

### Sequence assembly and translation

After adaptor filtering using Trimmomatic v0.33 [105] with default settings and retaining reads greater than 80 bp for 100 bp-length sequencing runs and 100 bp for 150 bp-length sequencing runs, we assembled all de novo transcriptomes using Trinity v2.0.6 [106] with default parameters (Trinity v2.3 was used for *H. auricula*, *L. quadricornis* and *C. borealis*). For each transcriptome, transcripts were translated into peptides using default settings in TransDecoder v2.0.1 [68]. We generated an assembly of the *Renilla reniformis* nuclear genome from Illumina paired-end reads as follows: we trimmed adapters with Trimmomatic v0.32 [105], performed error-correction with Allpaths-LG version 44,837 [107] and assembled the processed reads using Platanus version 1.2.1 (with default parameters except k = 48) [108]. We created a coding-region training set using the JGI genome annotations of *Nematostella vectensis* v1.0 [48] and then used Augustus 3.0.3 [109] with default parameters to generate *Renilla reniformis* protein predictions.

To minimize the possibility of integrating contaminant or laterally transferred sequences, we removed all sequences that had better BLAST [110] hits to outgroups than to ingroups. We did this in two steps: first against a database that included a representative set of metazoan and non-metazoan sequences (http://ryanlab.whitney.ufl.edu/downloads/alien_index/), and then against a database that included a set of representative cnidarian sequences and a set of representative bilaterian sequences (in GitHub repository). We used alien_index version 3.0 [111] to identify sequences with better hits to each outgroup and the remove_aliens script from the alien_index package to build a new FASTA sequence file that excluded potential contaminants. While this process likely removed numerous non-contaminant/non-laterally transferred sequences, our conservative approach made it less likely that we included contaminant sequences and the loss of data was acceptable given the great number of sequences that passed our conservative filter. All commands and scripts used for sequence assembly and translation are given in Additional file 13 and at https://github.com/josephryan/2017-Kayal_et_al.

### Construction of phylogenomic datasets

We built two preliminary datasets consisting of 54 cnidarian taxa and eight outgroups using *1)* Agalma v0.5-devel with nucleotide sequences as input (https://bitbucket.org/caseywdunn/agalma) as in Zapata et al. [10] and, *2)* a custom phylogenomics pipeline consisting of OrthoFinder v0.4.0 [46] followed by PhyloTreePruner [47] and our associated wrapper scripts that we refer to as OF-PTP. OF-PTP takes the TransDecoder-translated peptide sequences from each transcriptome as input. The final supermatrices produced by both approaches were filtered to include partitions with greater than 50% taxon occupancy, which were then used for phylogenetic analyses. Following preliminary phylogenetic analyses we noticed that the myxozoan taxa showed evidence of contamination even after extensive filtering with alien_index and this contamination resulted in Endocnidozoa being placed within Verterbrata in the Agalma, but not the OF-PTP dataset. To investigate this further, we obtained all data partitions from AG_62tx and OF_PTP_62tx that had myxozoan data and assessed how many putative contaminant sequences were present in each. We also conducted BLAST [110] similarity searches for each myxozoan sequence captured by both pipelines against a BLAST database comprised of the protein models from ten high-quality, phylogenetically disparate metazoan genomes, including teleosts and cnidarians. The frequency that the top BLAST hit for each myxozoan sequence resided in a data partition with one, two, three or four other myxozoan species was determined for each data matrix using custom scripts. In addition, while analyzing our preliminary datasets, our efforts and additional publications made available transcriptomic data for several additional cnidarian taxa. We therefore created a final dataset using the OF-PTP pipeline (OF-PTP_75tx) that included 13 of these taxa.

We estimated overlap in data composition between the OF-PTP and Agalma matrices by directly comparing the complement of *N. vectensis* sequences present in each data partition. To do this, we first cross referenced the *N. vectensis* sequences from each partition for each dataset to their full length protein model in the *N. vectensis* v1.0 genome release [48] using BLAST [110]. We then compared the single best hits for each partition recovered from each dataset. We also explored possible differences in the functional classes represented by sequences present in each data matrix by assigning GO terms to the *N. vectensis* sequences recovered by each pipeline, and to the global *N. vectensis* protein models, using Interproscan v5 [112]. GO terms were summarized using REVIGO [113] and their relative enrichment and/or depletion compared to the *N. vectensis* v1.0 protein models [48] was assessed using Fisher’s Exact Tests in custom R scripts. In addition, we determined the number of partitions that included data-per-taxon (taxon occupancy) in our final supermatrices using custom R scripts. Commands and scripts used in the construction of phylogenomic datasets can be found at https://github.com/josephryan/2017-Kayal_et_al.

### Phylogenetic analyses and character mapping

For all datasets, preliminary phylogenetic analyses were conducted under the Maximum Likelihood (ML) framework with the best-fit model (PROTGAMMAAUTO) on a single partition using RAxML v8 [114]. In addition, we estimated an ML tree for our final dataset (OF-PTP_75tx) using the partitioning scheme predicted by PartitionFinder2 [115]. For all ML analyses, we first performed 20 independent runs using random starting trees under the best-fit model (preliminary analyses) or the modeling scheme predicted by PartitionFinder2 and from these, chose the best scoring tree. In addition, for each analysis, we generated 500 bootstrap replicates under the cognate model as a measure of nodal support. We also conducted Bayesian analyses of OF-PTP_75tx by running two independent chains with PhyloBayes MPI v.1.6 [116] under the CAT-GTR model. Each chain was run for more than 4000 cycles and the resulting topologies were summarized using bpcomp with a burn-in of 0.25 and sampling every 10 trees using PhyloBayes v.4.1. The independent chains did not converge due to the position of the outgroup *Trichoplax adhaerens*. To estimate convergence for the cnidarian ingroup, we removed *T. adhaerens* from all sampled trees using the prune function in Phyutility [117] and reran bpcomp on both chains.

We conducted character-mapping analyses under the explicit statistical models for character evolution described in SIMMAP and implemented in phytools [53, 54, 55]. SIMMAP uses stochastic character mapping to simulate the evolution of characters on a posterior distribution of trees, resulting in estimates of Posterior Probability (PP) for the presence or absence of each trait at each node. We scored each taxon for presence or absence of photosynthetic endosymbionts (including *Symbiodinium* and zoochlorellae), colonial body plan, a medusa stage, and a polyp stage as discrete characters (Fig. 6). In addition, we estimated the rate of gain or loss of each character under a two-rate Markov process using corHMM [57] and, using these estimated rates, we then estimated the marginal likelihoods of single vs. multiple origins for each trait using indorigins [56, 118]. To test the robustness of analyses of independent origins to differences in rate parameters we also conducted these analyses with arbitrary, exaggerated rate parameters (Table 1). Commands and R scripts used for phylogenetic analyses and character mapping can be found at https://github.com/josephryan/2017-Kayal_et_al.

## Additional files

Additional file 1: Table of taxa and data sources used in the present study including information on pre- and post-filtering dataset sizes. (XLSX 19 kb)

Additional file 2: Table of geographic localities sampled in the present study. (XLSX 9 kb)

Additional file 3: Comparison of preliminary results from phylogenetic reconstruction of the OF-PTP_62tx and AG_62_tx datasets. Red circles at tips represent the number of data partitions present per taxon. The position of the myxozoan taxa in each dataset is indicated in yellow. (PDF 541 kb)

Additional file 4: Exploratory phylogenetic estimation of the 47 AG_62tx partitions that had greater than three myxozoan species present in each. For this preliminary analysis a single partition was run under the LG model in RAxML v 8.0 [19]. (PDF 294 kb)

Additional file 5: Data occupancy mapped onto ML phylogeny for OF_PTP_62tx. (PDF 288 kb)

Additional file 6: Data occupancy mapped onto ML phylogeny for AG_62tx. (PDF 3275 kb)

Additional file 7: GO analyses of *N. vectensis* complements from phylogenomic datasets analyzed, including the molecular function and biological processes categories. For each GO category, functional classes are annotated by color and their relative enrichment (green) or depletion (red) is given in heatmaps left of the GO term description. Inset describes dataset order for heatmaps. (PDF 619 kb)

Additional file 8: Data occupancy mapped onto ML phylogeny for OF-PTP_75tx. (PDF 1352 kb)

Additional file 9: ML analyses of the partitioned final data matrix OF-PTP_75tx with bootstrap support. (PDF 99 kb)

Additional file 10: Bayesian MCMC analyses of the concatenated OF-PTP_75tx dataset run under CAT-GTR. (PDF 327 kb)

Additional file 11: Table of taxonomic distributions and taxon occupancy comparing the current and previous phylogenomic studies of Cnidaria. (XLSX 9 kb)

Additional file 12: Extended materials and methods including information on nucleic acid extraction and sequencing. (PDF 82 kb)

Additional file 13 Supplementary command lines. Computer code used to execute all analyses. See also https://github.com/josephryan/2017-Kayal_et_al. (PDF 122 kb)

## Abbreviations

GO: Gene ontology
ML: Maximum Likelihood
PP: Posterior Probability
SRA: Sequence Read Archive
