# Supplementary figures and images for: Phylogenomics provides a robust topology of the major cnidarian lineages and insights on the origins of key organismal traits
Source: BMC Evol Biol. 2018 Apr 13;18:68. doi: 10.1186/s12862-018-1142-0 (PMC5932825; doi:10.1186/s12862-018-1142-0)

# OF-PTP\_62tx

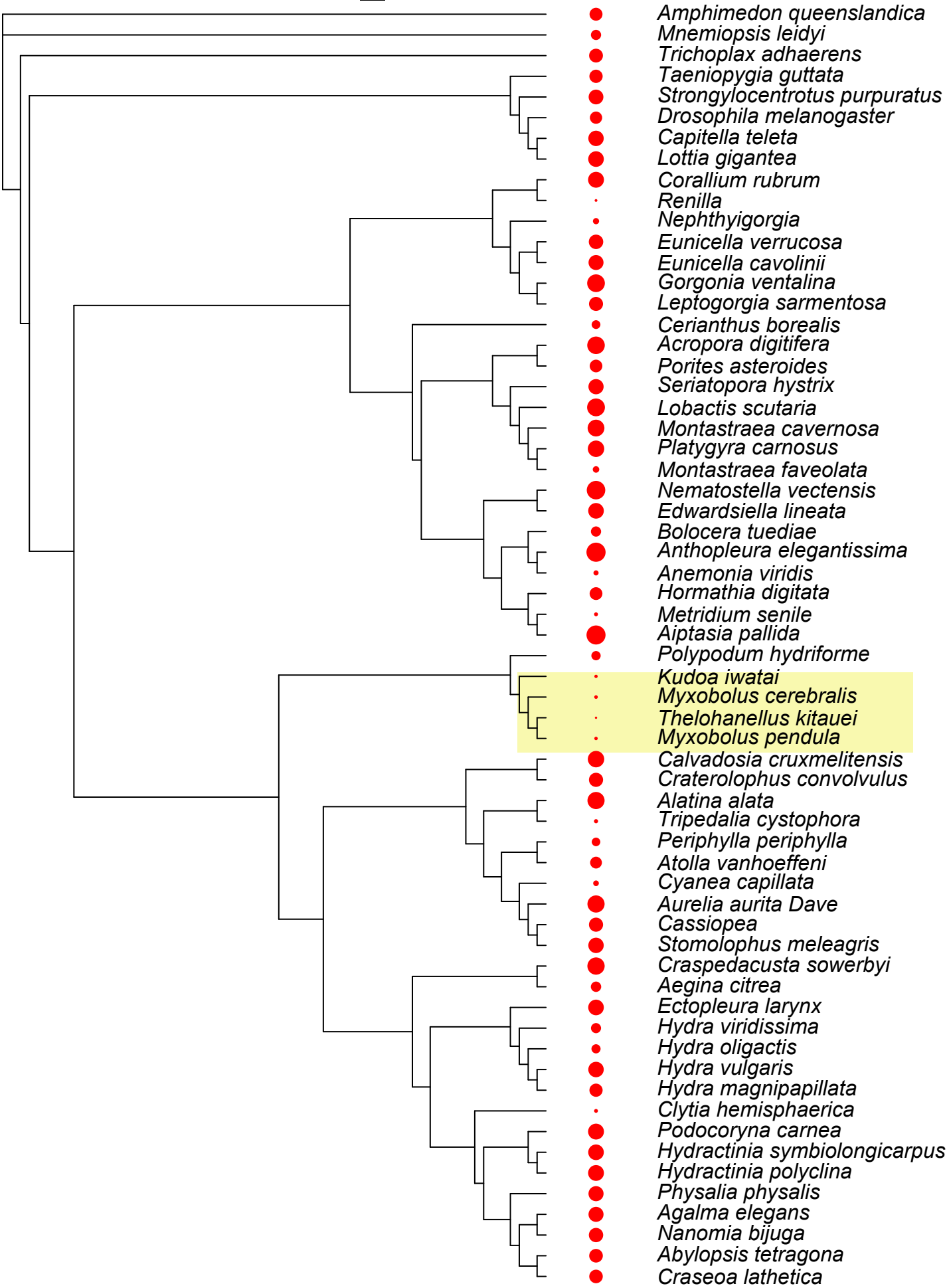

# AG\_62\_tx

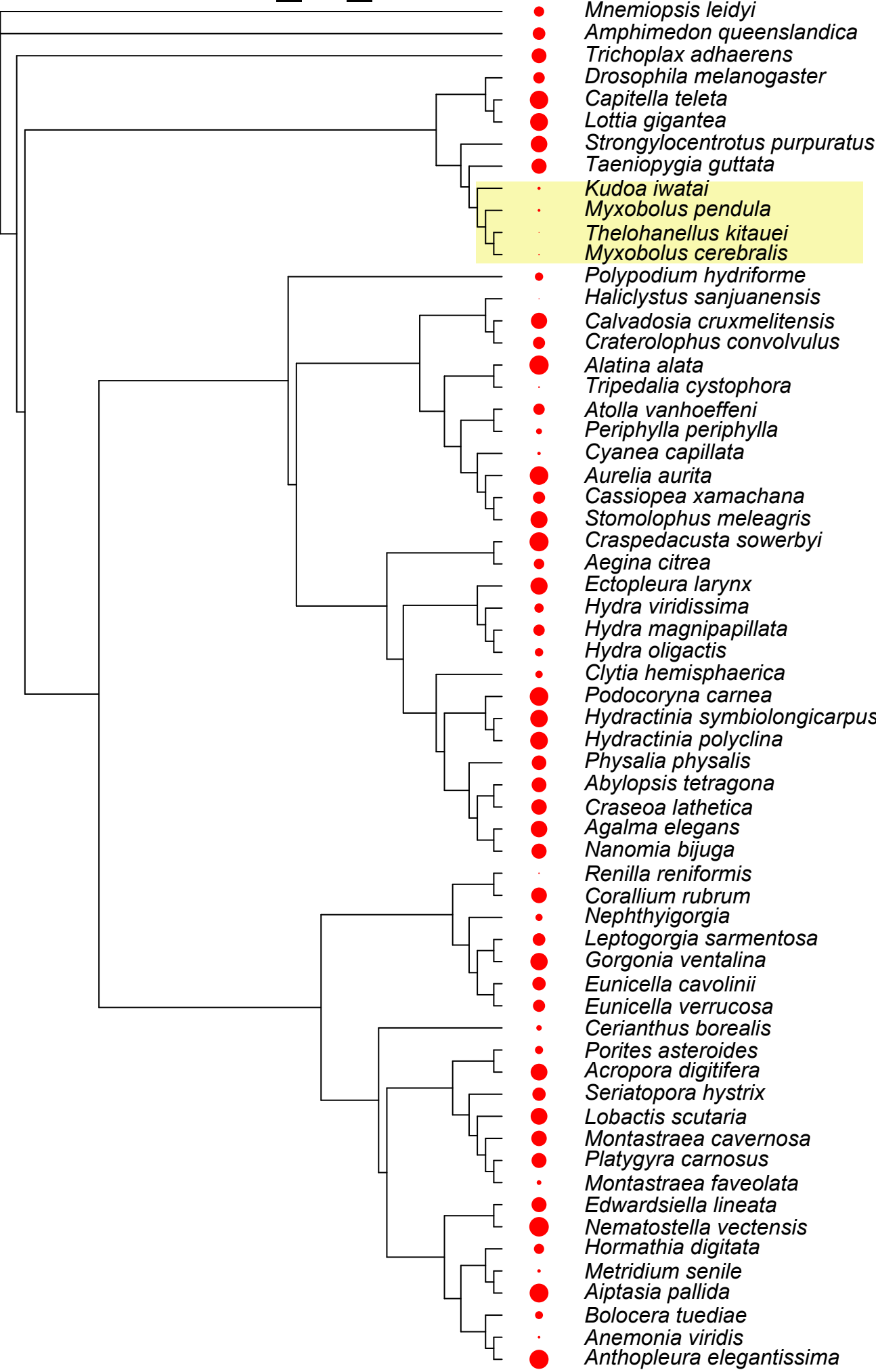

Supplement: Supplementary file 3 — Comparison of preliminary results from phylogenetic reconstruction of the OF-PTP_62tx and AG_62_tx datasets. Red circles at tips represent the number of data partitions present per taxon. The position of the myxozoan taxa in each dataset is indicated in yellow. (PDF 541 kb) [file 12862_2018_1142_MOESM3_ESM.pdf]

**Supplementary Figure S3**  
Data occupancy mapped onto ML phylogeny for OF\_PTP\_62tx

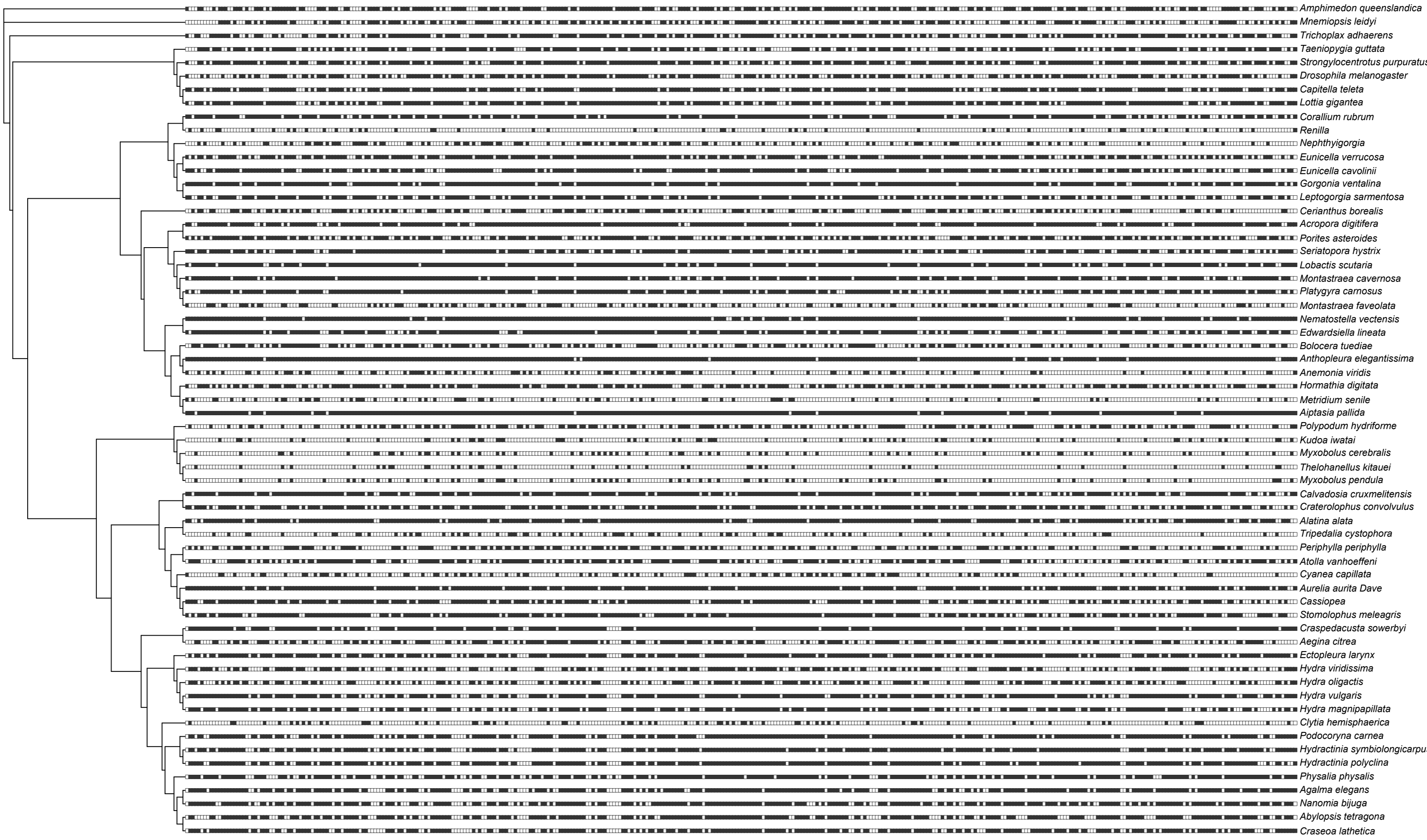

Supplement: Supplementary file 5 — Data occupancy mapped onto ML phylogeny for OF_PTP_62tx. (PDF 288 kb) [file 12862_2018_1142_MOESM5_ESM.pdf]

Supplementary Figure S4  
Data occupancy mapped onto ML phylogeny for AG\_62tx

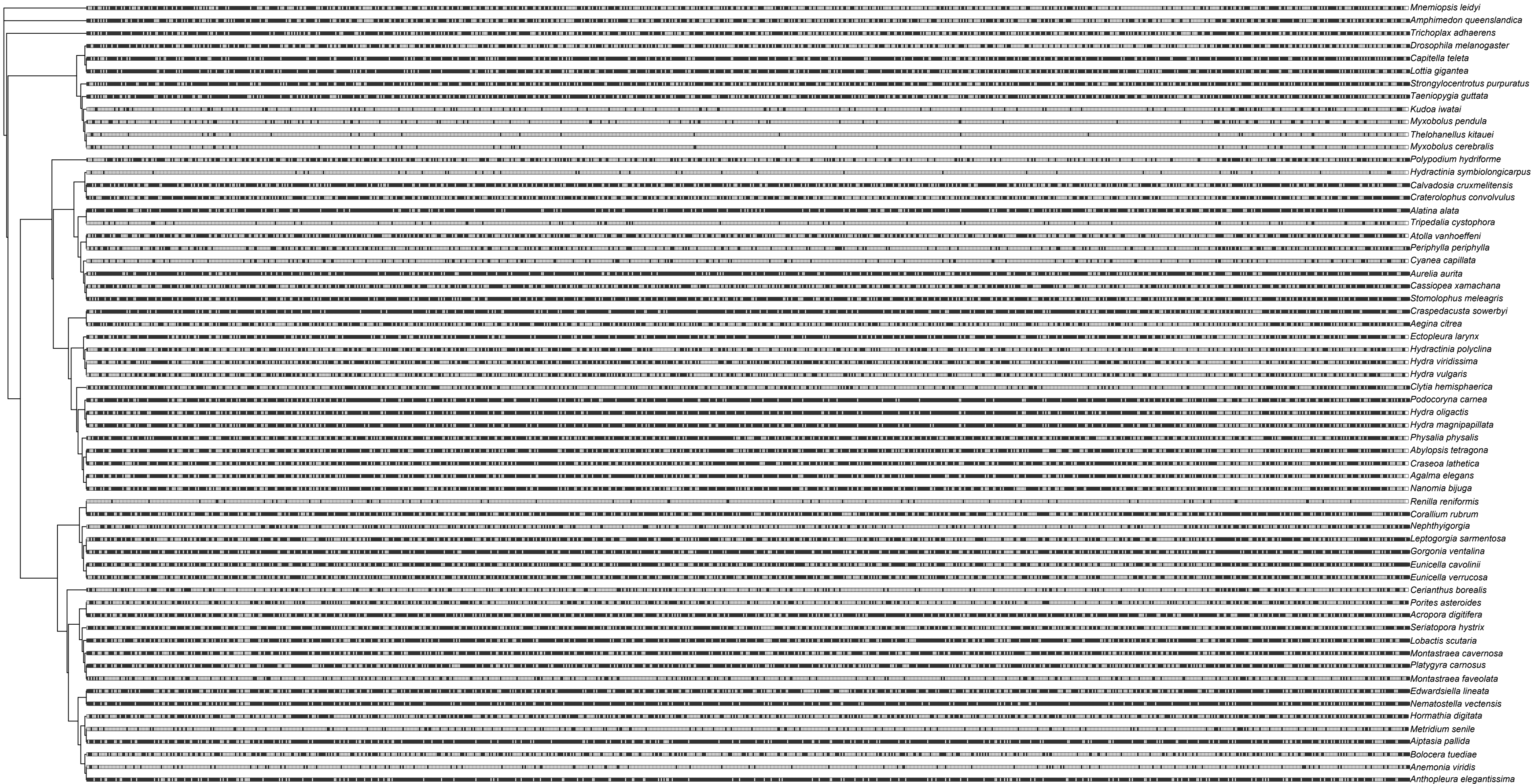

Supplement: Supplementary file 6 — Data occupancy mapped onto ML phylogeny for AG_62tx. (PDF 3275 kb) [file 12862_2018_1142_MOESM6_ESM.pdf]

**Supplementary Figure S6**  
Data occupancy mapped onto ML phylogeny for OF\_PTP+75tx

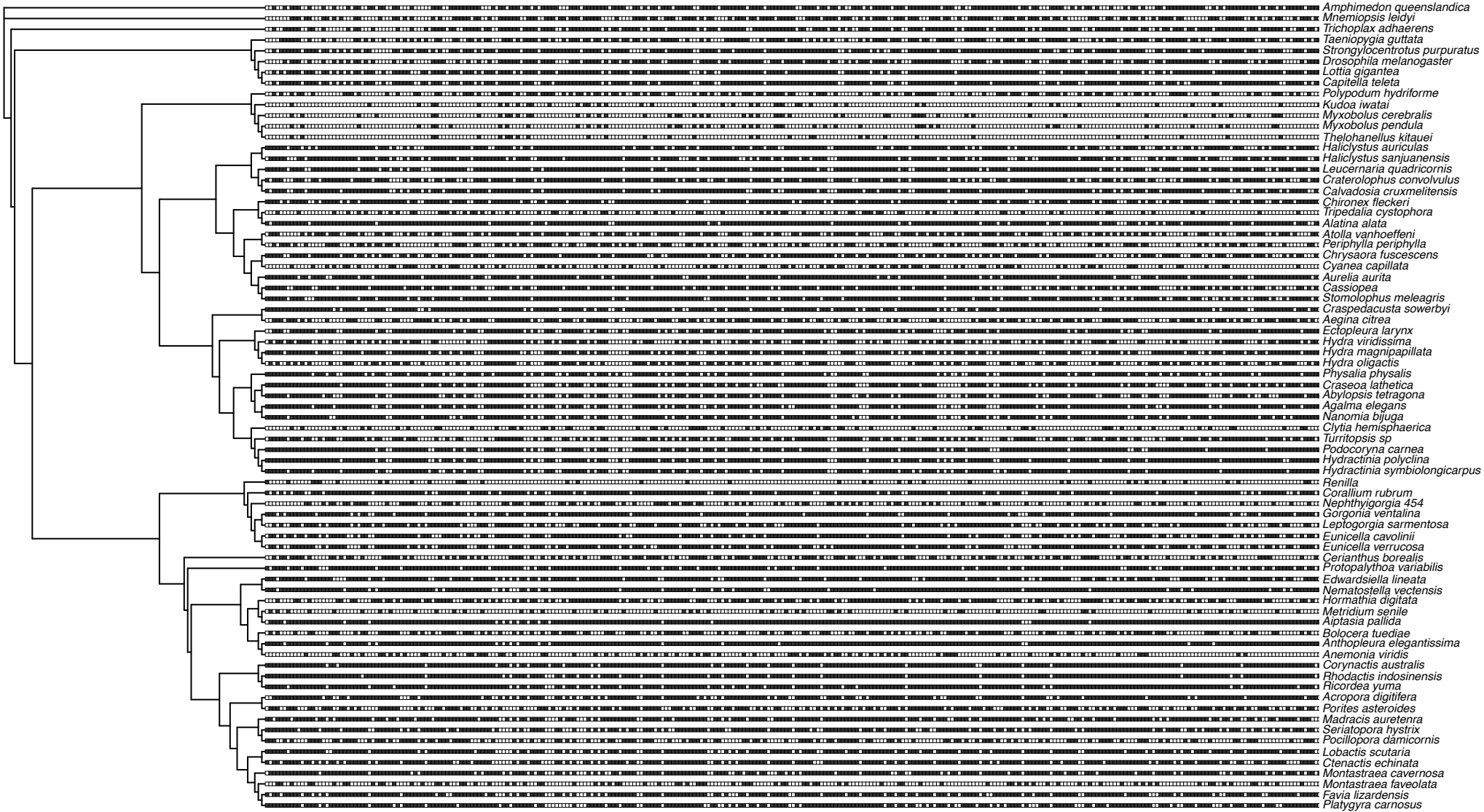

Supplement: Supplementary file 8 — Data occupancy mapped onto ML phylogeny for OF-PTP_75tx. (PDF 1352 kb) [file 12862_2018_1142_MOESM8_ESM.pdf]
