# Supplementary material for: Phylogenomics provides a robust topology of the major cnidarian lineages and insights on the origins of key organismal traits
Source: BMC Evol Biol. 2018 Apr 13;18:68. doi: 10.1186/s12862-018-1142-0 (PMC5932825; doi:10.1186/s12862-018-1142-0)
Supplement: Supplementary file 4 — Exploratory phylogenetic estimation of the 47 AG_62tx partitions that had greater than three myxozoan species present in each. For this preliminary analysis a single partition was run under the LG model in RAxML v 8.0 [19]. (PDF 294 kb) [file 12862_2018_1142_MOESM4_ESM.pdf]

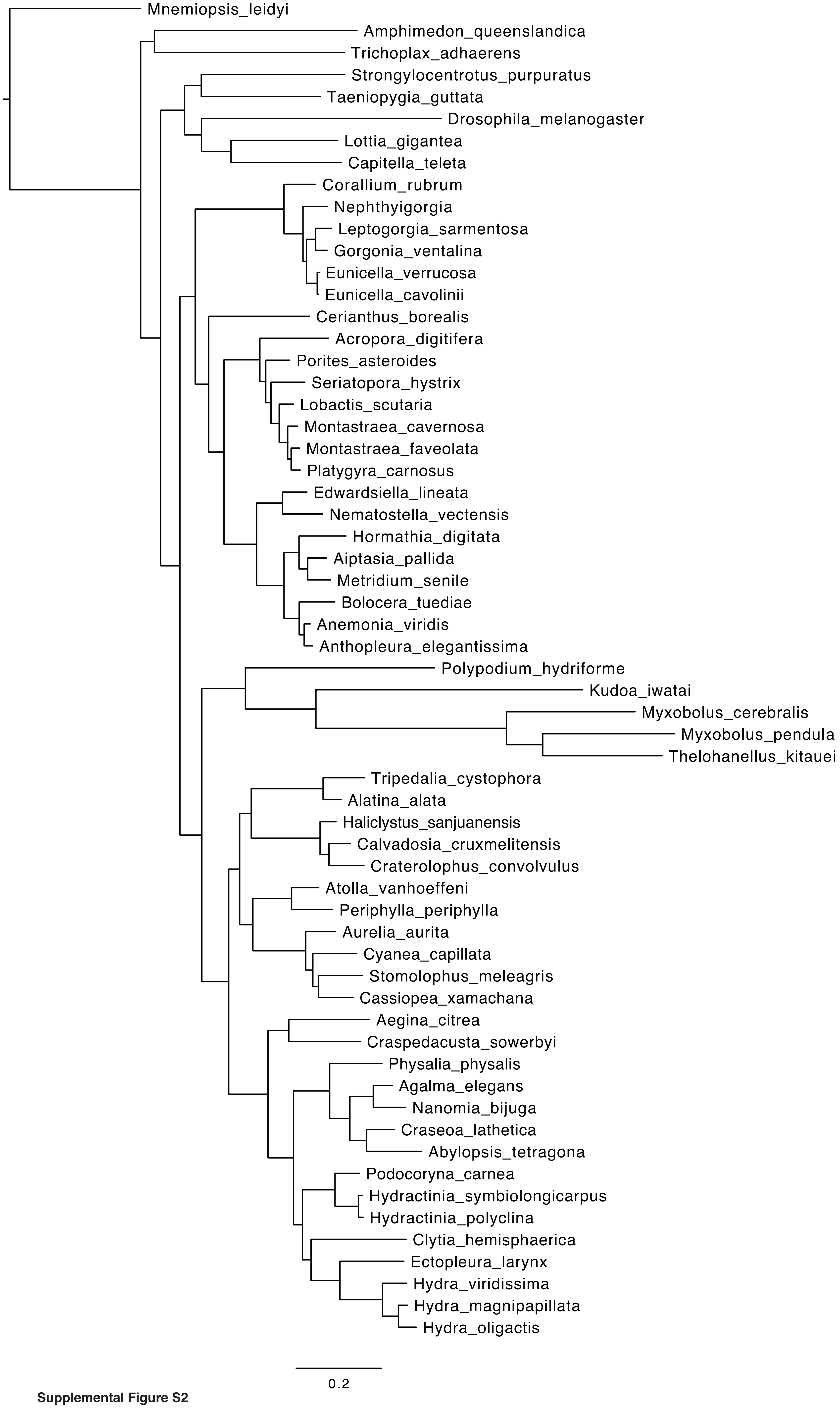

Supplemental Figure S2
Phylogenetic estimation of the 47 AG_62tx partitions that had greater than three myxozoan species present in each.
