# Supplementary material for: Phylogenomics provides a robust topology of the major cnidarian lineages and insights on the origins of key organismal traits
Source: BMC Evol Biol. 2018 Apr 13;18:68. doi: 10.1186/s12862-018-1142-0 (PMC5932825; doi:10.1186/s12862-018-1142-0)
Supplement: Supplementary file 7 — GO analyses of N. vectensis complements from phylogenomic datasets analyzed, including the molecular function and biological processes categories. For each GO category, functional classes are annotated by color and their relative enrichment (green) or depletion (red) is given in heatmaps left of the GO term description. Inset describes dataset order for heatmaps. (PDF 619 kb) [file 12862_2018_1142_MOESM7_ESM.pdf]

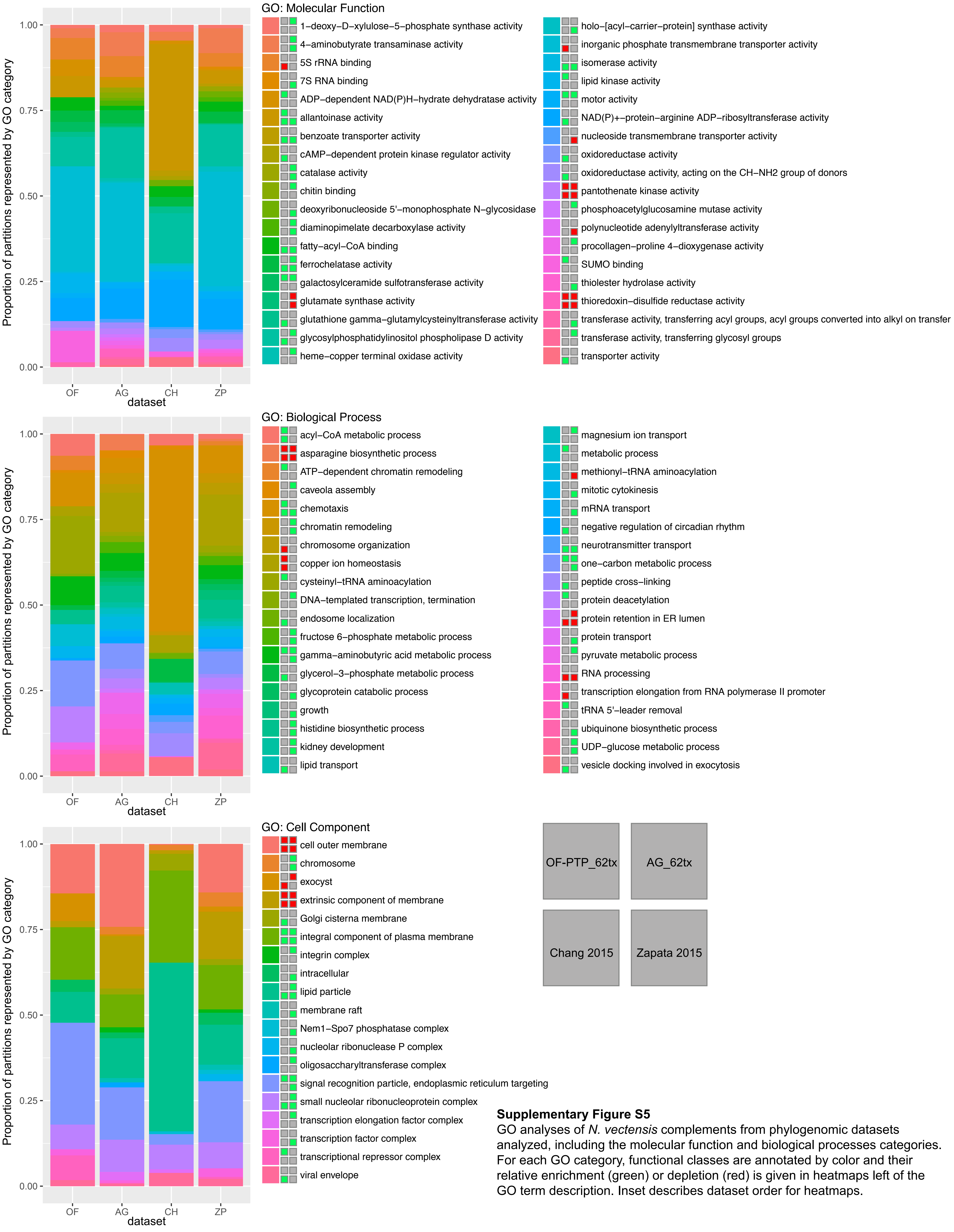

Supplementary Figure S5
GO analyses of *N. vectensis* complements from phylogenomic datasets analyzed, including the molecular function and biological processes categories. For each GO category, functional classes are annotated by color and their relative enrichment (green) or depletion (red) is given in heatmaps left of the GO term description. Inset describes dataset order for heatmaps.
