# Supplementary material for: Phylogenomics provides a robust topology of the major cnidarian lineages and insights on the origins of key organismal traits
Source: BMC Evol Biol. 2018 Apr 13;18:68. doi: 10.1186/s12862-018-1142-0 (PMC5932825; doi:10.1186/s12862-018-1142-0)
Supplement: Supplementary file 10 — Bayesian MCMC analyses of the concatenated OF-PTP_75tx dataset run under CAT-GTR. (PDF 327 kb) [file 12862_2018_1142_MOESM10_ESM.pdf]

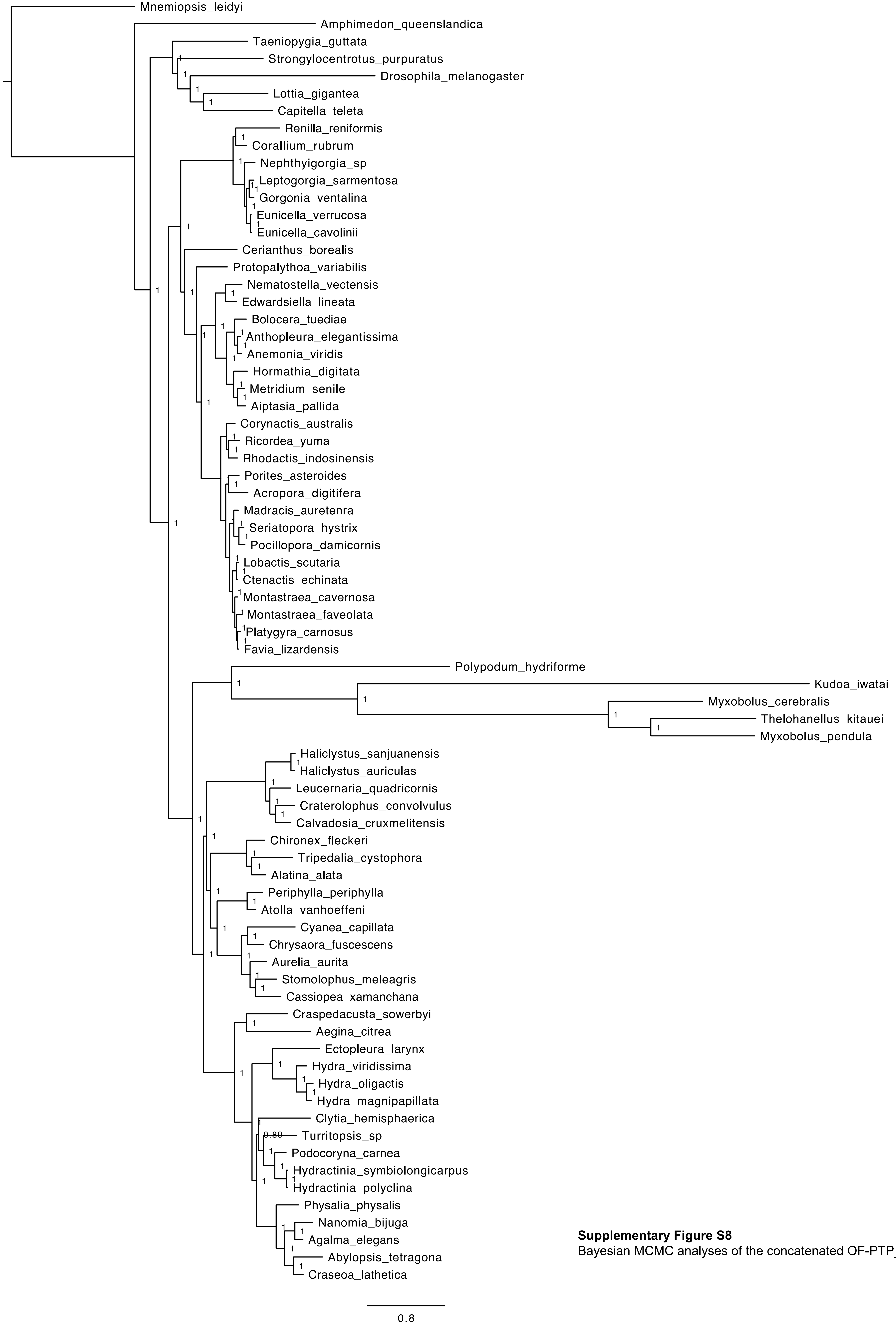

Supplementary Figure S8
Bayesian MCMC analyses of the concatenated OF-PTP_75tx dataset run under CAT-GTR
