# Supplementary material for: Phylogenomics provides a robust topology of the major cnidarian lineages and insights on the origins of key organismal traits
Source: BMC Evol Biol. 2018 Apr 13;18:68. doi: 10.1186/s12862-018-1142-0 (PMC5932825; doi:10.1186/s12862-018-1142-0)
Supplement: Supplementary file 12 — Extended materials and methods including information on nucleic acid extraction and sequencing. (PDF 82 kb) [file 12862_2018_1142_MOESM12_ESM.pdf]

Extended material and methods

DNA extraction

After months in the lab, one *Renilla reniformis* individual was divided into multiple pieces and placed in a 1.5 ml tube and covered with DNA extraction buffer (10mM Tris pH 8; 0,5 % SDS). The samples were coarsely grinded using a propylene pestle (Sigma, *Z359947*). A volume of RNase cocktail (Ambion, AM2286), equal to 1/10 of the original DNA buffer volume, was added to each sample and incubated at 37°C for an hour. Subsequently, proteinase K was added to the solution to a final concentration of 1µg/µl. The samples were incubated for an additional three hours at 50°C with gentle rocking, and then were centrifuged for one minute at 2000 rpm to pellet undigested debris. The supernatant was transferred to a fresh tube and went through three rounds of phenol:chloroform:isoamyl alcohol (25:24:1) purification steps. The DNA was then precipitated from the aqueous solution by adding 0.1 volumes of 3M Ammonium Acetate and 2.5 volumes of absolute Ethanol. The DNA was qualitatively analyzed in an agarose gel and quantified with a Nanodrop.

RNA extraction

For *C. cruxmelitensis*, the specimen was grinded in liquid nitrogen and mRNA extraction was performed with Dynabeads® mRNA DIRECT™ Purification Kit (ThermoFischer, *61011*) according to the manufacturer's manual using the standard protocol. For the *H. sanjuanensis* samples, mRNA was also extracted using the Dynabeads® mRNA DIRECT™ Purification Kit (ThermoFischer, *61011*) using either the micro, mini or standard protocols depending upon the size (i.e. life history stage) of the sample and according to the manufacturer's manual. mRNA extracts were quantified using the Agilent 2100 Bioanalyzer system with the mRNA Pico protocol and visualized on a High Sensitivity RNA ScreenTape with Agilent 2200 TapeStation system (Agilent Technologies). For *C. borealis*, *H. auricula*, *C. convolvulus* and *L. quadricornis*, individuals were dissected and samples were immediately homogenized in Trizol (Invitrogen) and total RNA was extracted using the PureLink RNA mini kit (Invitrogen). RNA quality and concentrations were assessed by agarose gel electrophoresis and a Qubit 2.0.

For *C. xamachana,* apo-symbiotic polyps were originally collected from the Key Largo, FL and raised in the laboratory for 2+ years. Polyps were maintained at 26 degrees C in 0.2 µm filtered artificial seawater. RNA was extracted from both apo-symbiotic and symbiotic polyps 3 and 8-days post-infection, as well as strobilas. Polyps infected with *Symbiodinium microadriaticum* (Clade A1) at a concentration of 10e-6 with newly hatched *Artemia*. Infected

polyps were kept at a 12 hour light-dark cycle at 150 mmol quanta $m^{-2}s^{-1}$. Polyps were unfed for 72 h prior to the 0, 3, and 8-day collection. Strobilas were collected during strobilation when the developing ephyra was noticeable, but prior to the start of pulsation. Polyps were preserved in RNAlater (ThermoFischer, AM7021) and stored at -80 degrees prior to processing. RNA was extracted using a standard phenol-chloroform extraction, with an overnight concentration step using lithium chloride. mRNA was purified from totalRNA using the Dynabeads® mRNA Purification Kit (ThermoFischer, *61006*) according to the manufacturer's manual using the standard protocol. mRNA extracts were quality was assessed using the Agilent 2100 Bioanalyzer system (Agilent Technologies) with the mRNA Nano protocol. Sequencing libraries were prepared following the RNAseq protocol from the Joint Genome Institute.

Library Preparation

For *Renilla reniformis*, DNA library preparation and sequencing were performed at the University of Florida Interdisciplinary Center for Biotechnology Research (USA). For *C. cruxmelitensis* and *H. sanjuanensis*, multiplexed RNA sequencing libraries were constructed using the ScriptSeq™ v2 RNA-Seq Library Preparation Kit (Epicentre, *SSV21124*) and ScriptSeq Index PCR Primers Set 1 (Epicentre, *SSIP1202*), respectively, following the manufacturer's protocol. *H. sanjuanensis* multiplexed libraries were pooled to an equimolar quantity. For *C. borealis*, *H. auricula*, *C. convolvulus* and *L. quadricornis*, RNA sequencing libraries were constructed using the NEBNext® Ultra RNA Library Prep Kit (NEB, E7530L) with NEBNext® Multiplex Oligos for Illumina® (Index Primers Set 1) (NEB, E7335S) following the manufacturer's protocol. Library quality was assessed using a High Sensitivity DNA ScreenTape with the Agilent 2200 TapeStation system (Agilent Technologies) and quantified with a Qubit dsDNA HS Assay Kit (ThermoFisher, *Q32854*) on a Qubit 2.0 system (Invitrogen) and an NEBNext® Library Quant Kit for Illumina (NEB, *E7630S*) on a ViiA™ 7 Real-Time PCR System (Applied Biosystems).

Library Sequencing

Renilla libraries were sequenced on an Illumina NextSeq 500 at the University of Florida's Interdisciplinary Center for Biotechnology Research (FL, USA). Libraries from *H. sanjuanensis* and *C. cruxmelitensis* were sequenced on an Illumina MiSeq machine with a V2 kit (2x300 cycles) at the Smithsonian Institution Laboratory of Analytic Biology (DC, USA). RNAseq libraries for *C. borealis*, *H. auricula*, *C. convolvulus* and *L. quadricornis* were pooled to equimolar ratios after quantification with a dsDNA HS Assay Kit (ThermoFisher, *Q32854*) on a

Qubit 2.0 system (Invitrogen) and sequenced on one lane of an Illumina HiSeq2500 machine at the Hubbard Center for Genome Sciences at the University of New Hampshire (NH, USA).
