# Supplementary material for: Phylogenomics provides a robust topology of the major cnidarian lineages and insights on the origins of key organismal traits
Source: BMC Evol Biol. 2018 Apr 13;18:68. doi: 10.1186/s12862-018-1142-0 (PMC5932825; doi:10.1186/s12862-018-1142-0)
Supplement: Supplementary file 13 — Supplementary command lines. Computer code used to execute all analyses. See also https://github.com/josephryan/2017-Kayal_et_al. (PDF 122 kb) [file 12862_2018_1142_MOESM13_ESM.pdf]

Supplementary Methods: Commands and Parameters used in Analyses

All scripts and data noted are found at https://github.com/josephryan/2017-Kayal_et_al

A. Data preparation and filtering

A1. FASTA files for each taxon converted to peptide sequences in Transdecoder 2.0.2 and headers altered using seq_processing.sh.

Dependencies: Transdecoder 2.0.2 (Hass 2013)

```
./seq_processing.sh
```

A2. FASTA files for each taxon BLASTed against metazoan/non-metazoa database (alien_index step 1) for more details see: https://github.com/josephryan/alien_index

Dependencies: BLAST (Altschul et al 1990)

```
blastx -query myseqs.fa -db ai.fa -outfmt 6 -max_target_seqs 1000 -seg
yes \
        -evalue 0.001 -out myseqs_v_ai.blastx
alien_index --blast=myseqs_v_ai.blastx --alien_pattern=ALIEN_ >
myseqs.alien_index
remove_aliens myseqs.alien_index myseqs.fa > myseqs2.fa
```

A3. FASTA files for each taxon BLASTed against cnidarian/bilaterian database (alien_index step 2)

```
blastx -query myseqs2.fa -db bilat_ai.fa -outfmt 6 -max_target_seqs
1000 -seg yes \
        -evalue 0.001 -out myseqs2_v_bilat_ai.blastx
alien_index --blast=myseqs2_v_ai.blastx --alien_pattern=ALIEN_ >
myseqs2.alien_index
remove_aliens myseqs2.alien_index myseqs2.fa > myseqs3.fa
```

B. De novo phylogenomic matrix construction

B1. Orthofinder procedure on all data

Dependencies: Orthofinder v0.4.0 (Emms and Kelly 2015); PhyloTreePrunner (Kocot et al 2013)

- Setup OrthoFinder for external BLAST searches:

```
orthofinder.py -f DB_redo –p
```

- Run array BLAST command lines produced by the previous command.
- Create orthogroups

```
orthofinder.py -b DB/Results_Jul22/WorkingDirectory/ -t $NSLOTS
```

- Make gene trees with OF:

```
trees_for_orthogroups.py -b DB_redo/Results_Dec15/WorkingDirectory/ -t
$NSLOTS
```

B2. Selection by taxon occupancy criterion for OF–PTP

- Use Phylotreepruner to select monophyletic clades within orthogroups created by OF

```
mkdir pruned
for x in Alignments/*; do runphylotreepruner  Trees/`basename
${x%.fa}`_tree.txt 37  Alignments/`basename ${x%.fa}`.fa   0.5 u ;
done
mv Alignments/*pruned* pruned/
```

- Make multiple alignments for each pruned orthogroup using MAFFT

```
#align pruned OGs
parallel -j14 'mafft --auto {} > {.}_aln'  ::: ./pruned_0.7/*.fa
#trim alignments
parallel -j14 'gblocks_wrapper {}' ::: ./pruned_0.7/*_aln
rm *.htm
#remove gaps
perl -pi -e 's/\ //g' ./pruned_0.7/*gb
#cut off OG idenfier so that seqCat can concatenate
parallel -j14 'cut -f1 -d"|" {} > {.}_rename' ::: ./pruned_0.7/*gb
#concatenate all OGs into single nexus
ls ./pruned_0.7/*_rename > parts_list
seqCat.pl -dparts_list
```

B3. Agalma procedure on all data

- Catalog each species into an sqlite database. Example:

```
agalma catalog insert --id "Craterolophus_convolvulus" -p
Craterolophus_convolvulus.v1.cds --species "Craterolophus convolvulus"
--ncbi_id "37531" --itis_id "51545"
```

- Process the nucleotide sequences for each species catalogued earlier. Example:

```
agalma postassemble --id Craterolophus_convolvulus —external
```

- Load each of the processed data. Example:

```
agalma load --id Craterolophus_convolvulus
```

- Make all-by-all comparisons of all loaded sequences:

```
agalma homologize --id Cnidaria_8_2016 63 64 65 66 67 68 69 70 71 72
73 74 75 76 77 78 79 80 81 82 83 84 85 86 87 88 89 90 91 92 93 94 95
96 97 98 99 100 101 102 103 104 105 106 107 108 109 110 111 112 113
114 115 116 117 118 119 120 121 122 123 129
```

- Make multiple alignments and alignment filtering of each orthologous group

```
agalma multalign --id Cnidaria_8_2016
```

- Make gene trees for each alignment

```
agalma genetree --id Cnidaria_8_2016
```

- Prune trees

```
agalma treeprune --id Cnidaria_8_2016
```

- Make multiple alignments and alignment filtering of each orthologous group

```
agalma multalign --id Cnidaria_8_2016
```

- Create a supermatrix based on the taxon occupancy value of 50%

```
agalma supermatrix --id Cnidaria_8_2016   --proportion 0.50
```

C. Phylogenetic analyses

C1. PartitionFinder2 analysis of OF_PTP_75tx

```
python PartitionFinderProtein.py OF_50/ --raxml --rcluster-max 1000 --
rcluster-percent 10
```

C2. RAxML command line for unpartitioned analyses

```
raxmlHPC-PTHREADS-SSE3 -T $NSLOTS -m PROTGAMMAAUTO  -p $RANDOM -# 20 -
s cnidOF_75_taxa.phy -n RAxMLbesttreeAUTO_OF75tx_unpart
```

```
raxmlHPC-PTHREADS-SSE3 -T $NSLOTS -m PROTGAMMALG -b $RANDOM -p $RANDOM
-#500 -s  cnidOF_75_taxa.phy  -n RAxMLbootOF75tx_unpart
```

```
raxmlHPC-PTHREADS-SSE3 -T $NSLOTS -m PROTGAMMALG -f b -z
boot/RAxML_bootstrap.RAxMLbootOrthofinder75tx_unpart -t
ML/RAxML_bestTree.RAxMLbesttreeAUTO_Orthofinder75tx_unpart -n
RAxMLfinal-treeAUTO-bootLG_Orthofinder75tx_unpart
```

C3. RAxML command line for partitioned analyses

```
raxmlHPC-PTHREADS-SSE3 -T $NSLOTS -m PROTGAMMAAUTO  -p $RANDOM -# 20 -
q part  -s cnidOF_75_taxa.phy -n RAxMLbesttreeAUTO_OF75tx_part
```

```
raxmlHPC-PTHREADS-SSE3 -T $NSLOTS -m PROTGAMMALG -b $RANDOM -p $RANDOM
-#500 -q  part  -s  cnidOF_75_taxa.phy  -n RAxMLbootOF75tx_part
```

```
raxmlHPC-PTHREADS-SSE3 -T $NSLOTS -m PROTGAMMALG -f b -z
RAxML_bootstrap.RAxMLbootOrthofinder75tx_unpart -t
RAxML_bestTree.RAxMLbesttreeAUTO_Orthofinder75tx_unpart -n RAxMLfinal-
treeAUTO-bootLG_Orthofinder75tx_unpart
```

C4. Phylobayes command line for OF_PTP_75tx

```
pb_mpi -d  cnidOF_75_taxa.phy -cat -gtr PhyloBayesCATGTRI_OF75tx_1
pb_mpi -d  cnidOF_75_taxa.phy -cat -gtr PhyloBayesCATGTRI_OF75tx_2
```

C5. tracecomp and bpcomp command line

```
tracecomp    PhyloBayesCATGTRI_OF75tx_2.treelist.pruned
PhyloBayesCATGTRI_OF75tx_1.treelist.pruned
```

```
bpcomp -x 1043 10 PhyloBayesCATGTRI_OF75tx_1.treelist.pruned
PhyloBayesCATGTRI_OF75tx_2.treelist.pruned
```

D. Post analyses of phylogenomic matrix composition

D1. Analysis of myxozoan partitions in OF-PTP_62tx and AG_62tx in R

Requires:
agalma.partitiontable.txt
OF50partitiontable.txt

```
Rscript Partition_comparison.R
```

D2. GO Analysis of matrices for Figure 4

Run NV sequences from each partition from each dataset through interproscan. Also run each NV peptide sequence from the NV 1.0 protein model through interproscan.

```
./all_scan.sh
```

Retain only unique GO terms with a e value greater than 0.001, write them to a new file

```
./select_GO_terms.sh
```

D3. The following R scripts were used for Figure 4.

Figure 4A

```
Rscript Overlay_3_23.R
```

Figure 4B

```
Rscript plot_mat.R
```

Figure 4C

```
Rscript venn.R
```

Figure 4C

```
Rscript NV_GO_Enrich_rels.R
```

E. Character mapping studies of cnidarian trait evolution

E1. Ancestral state reconstruction

Dependencies:
Phytools (Revell 2012)

Requires:
colonial_charmatrix.txt
medusa_charmatrix.txt
polyp_charmatrix.txt
symbiont_charmatrix.txt
Cnid_only.nex

```
Rscript Char_map_5.8.R
```

E2. Bayes factor tests of multiple origins

Dependencies:
Indorigin (Minin 2014)

Requires
colonial_charmatrix.txt
medusa_charmatrix.txt
polyp_charmatrix.txt
symbiont_charmatrix.txt
PhyloBayesCATGTRI_OF75tx_2.treelist.pruned

```
Rscript indorigin_dp.R
```

References

Altschul SF, Gish W, Miller W, Myers EW, Lipman DJ 1990. Basic local alignment search tool. J Mol Biol 215: 403-410. doi: 10.1016/S0022-2836(05)80360-2

Dunn CW, Howison M, Zapata F 2013. Agalma: an automated phylogenomics workflow. BMC Bioinformatics 14: 330. doi: 10.1186/1471-2105-14-330

Emms DM, Kelly S 2015. OrthoFinder: solving fundamental biases in whole genome comparisons dramatically improves orthogroup inference accuracy. Genome Biol 16: 157. doi: 10.1186/s13059-015-0721-2

Haas BJ, Papanicolaou A, Yassour M, et al. *De novo* transcript sequence reconstruction from RNA-Seq: reference generation and analysis with Trinity. *Nature protocols*. 2013;8(8):10.1038/nprot.2013.084. doi:10.1038/nprot.2013.084.

Kocot KM, Citarella MR, Moroz LL, Halanych KM 2013. PhyloTreePruner: A Phylogenetic Tree-Based Approach for Selection of Orthologous Sequences for Phylogenomics. Evol Bioinform Online 9: 429-435. doi: 10.4137/EBO.S12813

Minin VN SM, Imholte GC. 2014. indorigin: Testing how many times a trait of interest was regained during evolution (R package).

Revell LJ 2012. phytools: an R package for phylogenetic comparative biology (and other things). Methods in Ecology and Evolution 3: 217-223. doi: 10.1111/j.2041-210X.2011.00169.x
